# Advances and Challenges in Immunotherapy for Metastatic Uveal Melanoma: Clinical Strategies and Emerging Targets

**DOI:** 10.3390/jcm14145137

**Published:** 2025-07-19

**Authors:** Mariana Grigoruta, Xiaohua Kong, Yong Qin

**Affiliations:** 1 Department of Pharmaceutical Sciences, School of Pharmacy, The University of Texas at El Paso, El Paso, TX 79902, USA; mariana.grigoruta@uacj.mx (M.G.); xkong@miners.utep.edu (X.K.); 2 Department of Health Sciences, Biomedical Sciences Institute, Autonomous University of Ciudad Juarez, Ciudad Juárez 32310, Mexico

**Keywords:** metastatic uveal melanoma, immunotherapy, checkpoint inhibitors, TCR-based therapy, Tebentafusp, vaccines

## Abstract

Uveal melanoma (UM), the most common primary intraocular malignancy in adults, poses a unique clinical challenge due to its high propensity for liver metastasis and poor responsiveness to conventional therapies. Despite the expanding landscape of immunotherapy in oncology, progress in managing metastatic uveal melanoma (mUM) remains limited, and no universally accepted standard of care has been established. In this review, we examine the current state and evolving strategies in immunotherapy for mUM, focusing on immune checkpoint inhibitors (ICIs), T cell receptor (TCR)-engineered therapies, and tumor-targeted vaccines. We also present a meta-analytical comparison of clinical outcomes between ICI monotherapy and combination regimens, alongside the recently FDA-approved T cell engager tebentafusp. Our analysis indicates that the triple combination of Ipilimumab, anti-PD-1 agents, and tebentafusp significantly enhances objective response rates, disease control rates, 1-year overall survival rates, and median overall survival (mOS) compared to ICI monotherapy alone. However, this enhanced efficacy is accompanied by increased toxicity due to broader immune activation. In contrast, tebentafusp offers superior tumor specificity and a more favorable safety profile in HLA-A*02:01-positive patients, positioning it as a preferred therapeutic option for this genetically defined subset of UM. Additionally, early-phase studies involving dendritic cell-based immunotherapies and peptide vaccines has shown encouraging signs of tumor-specific immune activation, along with improved tolerability. Collectively, this review underscores the urgent need for more precise and effective immunotherapeutic approaches tailored to the unique biology of mUM.

## 1. Introduction

**Figure 1:**
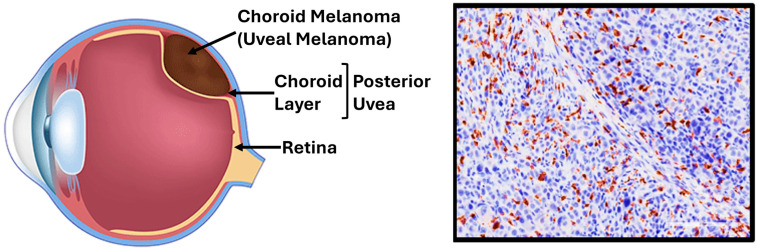
Anatomical illustration demonstrates a primary uveal melanoma (brown mass) arising from the choroid layer of the eye. (Right panel) Immunohistochemical analysis showing CD8+ T cell infiltration (red punctate staining) within the tumor microenvironment.

UM is the most common primary intraocular malignancy in adults, with the majority of cases diagnosed in individuals over the age of 60. The choroid is the predominant site of involvement, accounting for approximately 90% of UM cases [1,2] (Figure 1). At the time of diagnosis, most UM patients present with ocular symptoms and a localized tumor. However, despite appropriate tumor management, approximately 50% of patients eventually develop metastatic disease, often to the liver. The diagnosis of UM is primarily clinical, relying on slit-lamp examination and indirect ophthalmoscopy. Histological analysis plays a key role in prognostic assessment, with features such as tumor invasion into adjacent structures, inflammatory infiltrates, and elevated mitotic activity being associated with a poorer prognosis [3] (Figure 1).

Although UM accounts for only approximately 5% of all melanoma cases, it presents unique clinical and molecular features that distinguish it from cutaneous melanoma (CM), the most prevalent form. Both UM and CM originate from the malignant transformation of melanocytes, neural crest-derived pigment-producing cells. Melanocyte activity is regulated by various factors, including ultraviolet radiation (UVR) exposure, hormones and other mediators, and genetic mutations, all of which play critical roles in tumorigenesis, treatment response, and prognosis. While CM is strongly linked to UVR, UM develops in a UV-protected intraocular environment, suggesting divergent oncogenic pathways. Despite diagnosis and therapeutic advancements, melanoma remains a highly aggressive malignancy with a significant risk of metastasis and mortality, underscoring the need for improved management strategies [4].

The prognosis for patients with mUM remains poor, with limited therapeutic options and a median survival of less than 12 months following diagnosis [1]. Unlike CM, which has benefited from advances in immunotherapy [4], mUM is characterized by a lower tumor mutational burden, contributing to its resistance to ICIs and conventional chemotherapeutic agents [5]. Genomic alterations unique to UM include frequent activating mutations in GNAQ and GNA11. While these mutations have diagnostic relevance, they lack substantial prognostic value. In contrast, chromosomal aberrations, such as monosomy 3 and 8q amplification, as well as BAP1 mutations, are strongly associated with early metastasis and poor clinical outcomes [6]. Furthermore, epigenetic dysregulation, including altered DNA methylation changes, aberrant microRNA expression, and histone modifications, plays a key role in UM tumorigenesis and progression [7].

Melanin plays a dual role in melanoma pathogenesis, providing photoprotection through UV radiation absorption and antioxidant generation in normal physiology, while paradoxically promoting oncogenesis in melanoma. The melanogenesis process and associated driver mutations appear to facilitate tumor development through apoptosis inhibition, while reactive intermediates generated during pigment synthesis modify tumor metabolism and suppress immune responses, thereby enabling immune evasion. Furthermore, melanin contributes to therapeutic resistance against both immunotherapy and radiotherapy. Clinical evidence consistently demonstrates an inverse correlation between tumor pigmentation and prognosis, suggesting that melanin accumulation may represent an adaptive mechanism for tumor survival [8]. These findings support the hypothesis that melanogenesis inhibition could potentially enhance treatment efficacy in UM, warranting further investigation into the underlying molecular mechanisms and development of targeted therapeutic strategies against melanogenic pathways.

Currently, UM is managed primarily through resection, radiation therapy, and enucleation. Radiation therapy options include plaque brachytherapy using isotopes such as iodine-125, ruthenium-106, palladium-103, or cobalt-60, as well as teletherapy approaches like proton beam, helium ion therapy, or stereotactic radiosurgery (CyberKnife, GammaKnife, or linear accelerator-based systems). While enucleation serves as an alternative to radiation therapy, these methods demonstrate effective local tumor control. However, long-term patient survival remains concerning due to the high propensity for liver metastasis, which represents the major cause of mortality in UM patients [1,2].

Our group has shown that mUM tumors are enriched with immune cell infiltrates compared to primary lesions. Paradoxically, this immune-rich microenvironment does not correlate with increased sensitivity to ICIs and may instead reflect a state of immune dysfunction or exhaustion [9]. These findings underscore the need to understand and overcome the unique mechanisms of immune evasion in mUM.

Immunotherapy aims to restore or enhance cytotoxic T cell-mediated recognition and the destruction of tumor cells. While ICIs targeting CTLA-4, PD-1, and PD-L1 have transformed the management of CM and other malignancies, their efficacy in mUM has been limited. Response rates remain low, and durable clinical benefit is rare. Nevertheless, ongoing clinical efforts are investigating novel immunotherapeutic strategies, including cancer vaccines, dendritic cell-based therapies, and TCR-engineered approaches, which show promise in early-phase trials [10,11,12,13,14,15,16,17]. Among these, tebentafusp (IMCgp100), a bispecific TCR-based therapy targeting gp100 in HLA-A*02:01-positive patients, recently received FDA approval for mUM and represents a breakthrough in the field [18]. However, there is an urgent need for alternative therapies for non-eligible individuals.

In addition to systemic therapies, various locoregional and ablative modalities are being used to manage hepatic metastases in mUM [19]. These include hepatic resection (metastasectomy), isolated hepatic perfusion (IHP), and percutaneous hepatic perfusion (PHP) with melphalan chloride, transarterial chemoembolization (TACE), immunoembolization, and selective internal radiation therapy (SIRT) [20]. Melphalan, developed initially as a cytotoxic agent, has also demonstrated immunomodulatory properties that may contribute to its clinical benefit in mUM [21].

Oncogenesis represents a multifaceted biological process that disrupts systemic homeostasis through the dysregulation of neuroendocrine and immune networks. Tumors, particularly melanoma, actively participate in this dysregulation by secreting various bioactive molecules, including melanocyte-stimulating hormone, proopiomelanocortin, melatonin, catecholamines, and dopamine, while simultaneously expressing their corresponding receptors, which regulate both nervous and immune system functions. This bidirectional communication between tumor cells and host systems not only drives disease progression but also significantly influences therapeutic responses [22].

The clinical evaluation of melanoma requires the careful consideration of established prognostic factors such as patient age, tumor location, size, and extent of invasion. However, current clinical parameters demonstrate limited predictive capacity for disease behavior, highlighting the critical need for more reliable biomarkers. In UM, molecular markers including BRCA1-associated protein 1 (BAP1) mutations, preferential expressed antigen in melanoma (PRAME) expression, and characteristic chromosomal abnormalities (such as monosomy 3 and 8q amplification) have emerged as robust predictors of metastatic risk and treatment response. Recent comprehensive studies have further identified and validated additional molecular candidates that show promising prognostic potential [23].

This review aims to provide a comprehensive overview of the current immunotherapeutic landscape in mUM. We evaluate the efficacy of ICIs both as monotherapy and in combination regimens, as well as TCR-based therapies such as tebentafusp and investigational cancer vaccines. A key aspect of our review is the focus on emerging molecular targets and immunogenic candidates, which have the potential to shape future therapeutic development. We compile data from completed and ongoing clinical trials to contextualize progress and challenges in the treatment of mUM.

## 2. Current Immunotherapy for Patients with mUM

Immunotherapy has transformed the management of various malignancies by achieving durable responses in several tumor types. However, its impact on mUM has remained limited. Despite significant progress in the treatment of cutaneous melanoma, mUM continues to be associated with a poor prognosis, and, to date, no standardized therapeutic regimen has been established. Current treatment approaches typically involve systemic targeted agents, immune-based therapies, or liver-directed chemotherapeutics. Among immune-based strategies, current immunotherapeutic options for mUM can be categorized into three primary modalities: ICIs, TCR-based therapies, and vaccines. Vaccine-based strategies include dendritic cell (DC)-based, peptide-based, DNA-based, and viral vector-based approaches (Table 1).

In addition to these main categories, several other immunomodulatory agents are under clinical evaluation or have already entered clinical use, aiming to enhance anti-tumor immunity in mUM patients. In the following sections, we discuss the clinical efficacy and immunological mechanisms of each therapeutic category, with emphasis on patient responses, treatment limitations, and outcomes from recent clinical trials.

To further evaluate the therapeutic impact of currently available agents, we performed a focused statistical analysis comparing clinical outcomes of conventional ICI therapies with those of tebentafusp, a recently FDA-approved TCR-based drug. To identify relevant clinical trials, we conducted a comprehensive literature and database search between June and September 2022. Databases used included PubMed, Google Scholar, AACR Journals, ClinicalTrials.gov, and clinicaltrialsregister.eu. Search terms included “immunotherapy in uveal melanoma”, “intraocular melanoma”, “melanoma of the uvea”, “eye melanoma”, and “choroidal melanoma.” We included only clinical studies involving human mUM patients of any age and sex treated with ICIs (anti-CTLA-4, anti-PD-1, anti-PD-L1) or tebentafusp. Retrospective and prospective clinical studies were selected, provided they reported clearly distinguishable and complete data specific to mUM patients. Studies lacking key outcome metrics, data from non-metastatic patients, or mixed-melanoma cohorts without separation into the mUM subgroup were excluded from the analysis.

The final dataset included 21 studies on anti-CTLA-4 monotherapy, 21 studies on anti-PD-1 monotherapy, 16 studies on combination ICI therapy, and 3 studies on tebentafusp monotherapy (detailed in Supplementary Tables S1–S3). The clinical response categories analyzed were complete response (CR), partial response (PR), stable disease (SD), and progressive disease (PD), typically assessed based on the Response Evaluation Criteria in Solid Tumors (RECIST). However, some studies did not explicitly reference the criteria.

To assess treatment efficacy, we focused on key clinical endpoints. Objective response rate (ORR) was calculated as the sum of CR and PR cases, while disease control rate (DCR) included CR, PR, and SD cases. Overall survival (OS) was defined as the median time (in months) from treatment initiation to death due to UM, and progression-free survival (PFS) was defined as the time from treatment start to the radiologically confirmed disease progression.

In addition to the meta-analysis, we compiled all completed and ongoing clinical trials involving mUM immunotherapies registered in ClinicalTrials.gov. These studies are presented in chronological order based on their registry numbers, offering a timeline of research activity in the field and insights into the evolving therapeutic landscape of immunotherapy for mUM.

### 2.1. Immune Checkpoint Inhibitors (ICIs) in Patients with mUM

In the tumor immune landscape, CTLA-4 and PD-1 act as critical negative regulators of T cell activation. CTLA-4 competes with the costimulatory receptor CD28 for binding B7 molecules (CD80/86) on antigen-presenting cells, while PD-1 inhibits TCR signaling upon engagement with PD-L1 on tumor or immune cells. ICIs such as ipilimumab (anti-CTLA-4), nivolumab and pembrolizumab (anti-PD-1), and atezolizumab, durvalumab, and avelumab (anti-PD-L1) aim to restore T cell effector function by blocking these inhibitory interactions. ICIs have redefined cancer immunotherapy by reactivating suppressed T cell responses within the tumor microenvironment. These agents—primarily monoclonal antibodies targeting CTLA-4, PD-1, or PD-L1—are now integral in the treatment of several cancers, including CM, renal cell carcinoma, and non-small-cell lung cancer [81,82]. However, their efficacy in mUM remains limited, likely due to the unique immunobiology of this disease [83] (Table 2).

#### 2.1.1. Anti-CTLA-4 Therapy in mUM

**Figure 2:**
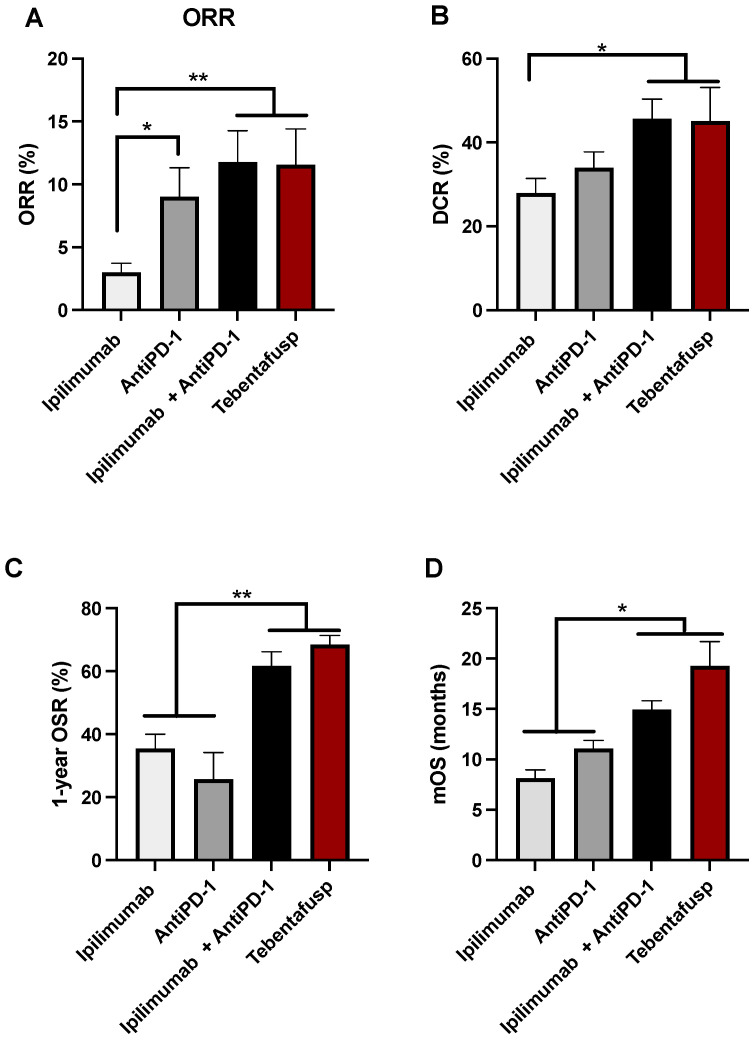
Comparative efficacy of ICIs and tebentafusp in mUM. Bar graphs depict clinical outcomes in mUM patients treated with monotherapy anti-CTLA-4 (Ipilimumab), monotherapy anti-PD-1 (nivolumab or pembrolizumab), combination anti-PD-1 and anti-CTLA-4, and tebentafusp. The outcomes analyzed include: (**A**) objective response rate (ORR), defined as the percentage of patients achieving complete or partial responses; (**B**) disease control rate (DCR), calculated as the proportion of patients with complete response, partial response, or stable disease; (**C**) one-year overall survival rate (1-year OSR); and (**D**) median overall survival (mOS), measured in months. The analysis included a total of 530 patients treated with Ipilimumab [24,25,26,27,28,29,30,31,32,33,34,35,36,37,38,39,40,41,42,43,44], 636 patients treated with anti-PD-1 agents (nivolumab and/or pembrolizumab [38,39,40,43,47,48,49,50,51,52,53,54,55,56,57,58,59,60,61], 595 patients treated with combination ICI therapy (anti-PD-1 + anti-CTLA-4) [40,49,64,65,66,67,68,69,70,71,72,73,74,75,76,77], and 397 patients treated with Tebentafusb [18,78,79,110] (see Supplemental Tables S1–S3 for study details). Data are presented as means ± standard error of the mean (SEM). Statistical significance is indicated as * *p* ≤ 0.05 and ** *p* ≤ 0.01.

Ipilimumab, the first ICI approved for advanced CM [82], has shown limited efficacy in mUM. Among the two CTLA-4 inhibitors tested, ipilimumab is the clinically preferred choice over tremelimumab. Only two studies have investigated tremelimumab in UM, reporting a mOS of 12.8 months and, when combined with IFNγ-2b, a DCR of 62.5% [45,46]. Our review of 22 studies on ipilimumab monotherapy revealed a modest DCR of 33.9% and a mOS of 8.1 months (Figure 2) [24,25,26,27,28,29,30,31,32,33,34,35,36,37,38,39,40,41,42,43,44]. Currently, no active clinical trials are evaluating CTLA-4 monotherapy in mUM, though it is commonly included in combination regimens (Table 2).

#### 2.1.2. Anti-PD-1/PD-L1 Monotherapy in mUM

PD-1 blockade has demonstrated slightly higher efficacy than CTLA-4 inhibition in mUM. Among PD-1 inhibitors, pembrolizumab was associated with better DCR than nivolumab (*p* ≤ 0.05), and the ORR was significantly higher in the PD-1 group than in the CTLA-4 group (*p* ≤ 0.05, Figure 2) [38,39,40,43,47,48,49,50,51,52,53,54,55,56,57,58,59,60,61]. These modest improvements in clinical outcomes underscore the need for further research and development in immunotherapy. Currently, two ongoing clinical trials (NCT03025256 and NCT04802876) are exploring anti–PD-1 monotherapy in mUM using nivolumab and spartalizumab.

Spartalizumab and LVGN3616 are monoclonal antibodies (mAbs) targeting the PD-1 receptor, designed to restore T cell-mediated anti-tumor activity. In patients with advanced BRAF(V600)-mutant melanoma, spartalizumab has been evaluated in combination with BRAF and MEK inhibitors. This regimen demonstrated only modest clinical efficacy and was associated with an increased incidence of treatment-related adverse events [84]. However, the field is dynamic and evolving, with ongoing trials investigating LVGN3616 in combination with a CD40 inhibitor Ab (LVGN7409) and/or a CD137 agonist Ab (LVGN6051) (NCT05075993).

#### 2.1.3. Combination ICI Therapy in mUM

Dual immune checkpoint inhibition using anti-CTLA-4 and anti-PD-1 agents has shown improved efficacy over monotherapy. Our meta-analysis confirms that combination therapy enhances ORR, DCR, 1-year OSR, and mOS in mUM (Figure 2). For example, a meta-analysis of eight studies (n = 379) reported an ORR of 13.7%, a CR rate of 2.1%, and a mOS ranging from 12.7 to 19.1 months, substantially better than outcomes observed with monotherapy [85].

Notably, a phase II trial combining ipilimumab and nivolumab with melphalan-based percutaneous hepatic perfusion (PHP) reported preliminary ORR of 85.7%, DCR of 100%, and median progression-free survival (mPFS) of 22.4 months [56,57]. Though not included in our pooled analysis due to small cohort size, these findings highlight the potential of integrated multimodal approaches.

Additionally, combining ICIs with cytokine-based therapies such as interleukin-2 (IL-2) and granulocyte–macrophage colony-stimulating factor (GM-CSF) has shown partial efficacy in stabilizing metastatic liver disease in UM. In one study, treatment with ipilimumab in conjunction with IL-2 and GM-CSF via immunoembolization resulted in disease stabilization in 72% of patients. However, disease progression was observed in 44% of cases by day 185.5, indicating a transient benefit limited to a subset of patients [86]. Multiple ongoing clinical trials are currently investigating various ICI-based combination strategies for mUM, reflecting the continued effort to improve clinical outcomes through synergistic immunomodulatory approaches (Table 2).

#### 2.1.4. Novel ICIs and Next-Generation Checkpoint Targets

Given the limited efficacy of conventional ICIs targeting PD-1, PD-L1, and CTLA-4 in mUM, there is an urgent need to explore novel immune regulatory pathways that contribute to tumor immune evasion. Several next-generation checkpoint molecules, including T cell immunoglobulin and mucin-domain containing-3 (TIM-3), lymphocyte activation gene-3 (LAG-3), and T cell immunoreceptor with Ig and ITIM domains (TIGIT), have emerged as promising therapeutic targets in UM and other solid tumors.

TIM-3, also known as hepatitis A virus cellular receptor 2 (HAVCR2), is expressed on both tumor and immune cells and acts as an inhibitory receptor that limits effector T cell responses. Its overexpression has been associated with poor clinical outcomes in several solid malignancies, including UM [87]. Notably, elevated TIM-3 expression has been observed in BAP1-deficient UM tumors, which are known to exhibit aggressive behavior and early metastatic progression [87,88]. In a recent first-in-human clinical trial (NCT03652077), the TIM-3–blocking monoclonal antibody INCAGN02390, an Fc-engineered human IgG1κ, demonstrated an acceptable safety profile and early evidence of efficacy in patients with advanced solid tumors, including UM. Among participants, the DCR was 17.5%, with one UM patient achieving a partial response lasting over 5.6 months [89].

LAG-3 is another co-inhibitory receptor expressed on tumor-infiltrating lymphocytes and tumor cells, where it acts to suppress T cell proliferation and function. In UM, increased LAG-3 expression has been correlated with poor prognosis and more aggressive disease behavior [90]. Relatlimab, a human IgG4 monoclonal antibody targeting LAG-3, was recently approved by the U.S. FDA in combination with nivolumab for the treatment of unresectable or metastatic melanoma. In a phase III clinical trial (NCT02519322), this combination therapy resulted in substantial clinical benefit, including a PR rate of 56%, SD in 38% of patients, and PD in only 6%, based on RECIST criteria [91]. These findings support the potential utility of LAG-3 inhibition in UM, particularly when used in combination with existing ICIs.

TIGIT, an inhibitory receptor expressed on T cells, natural killer (NK) cells, and regulatory T cells (Tregs), has also been implicated in tumor immune escape. It interacts with the poliovirus receptor (CD155) and CD112 on tumor and antigen-presenting cells to suppress anti-tumor immunity. In UM, TIGIT is frequently upregulated and contributes to immune suppression and resistance to immunotherapy. Its expression is often associated with increased infiltration of FOXP3+ CD4+ Tregs, which promote immunosuppressive tumor microenvironments and predict unfavorable outcomes in melanoma [92]. Thus, TIGIT represents a compelling target for future immunotherapeutic strategies in mUM, either alone or in combination with PD-1 or LAG-3 inhibitors.

Collectively, these novel immune checkpoints represent a promising avenue for expanding immunotherapy options in UM. Ongoing and future clinical trials targeting TIM-3, LAG-3, and TIGIT will help define their therapeutic potential and may provide a more effective and tailored approach to managing mUM.

#### 2.1.5. Immunomodulatory Agents and ICI Combinations

In addition to conventional ICIs, several immunostimulatory agents have been investigated for their potential to enhance anti-tumor immune responses, either as monotherapies or in combination with ICIs. Among these, SD-101 and PV-10 have emerged as promising candidates, particularly in melanoma, including mUM.

SD-101 is a synthetic CpG oligonucleotide that functions as a Toll-like receptor 9 (TLR9) agonist. It is designed to activate plasmacytoid dendritic cells and promote innate immune responses within the tumor microenvironment. In the PERIO-01 open-label phase I clinical trial (NCT04935229), SD-101 was administered via intratumoral injection into the liver metastases of UM patients using pressure-enabled hepatic artery infusion, in combination with systemic ipilimumab and nivolumab. The treatment was well tolerated and associated with immunologic activity suggestive of synergistic potential. Notably, even low doses of SD-101 combined with nivolumab elicited promising biologic effects, indicating that this approach may amplify T cell-mediated anti-tumor responses without inducing high-grade toxicity [93]. In previous studies involving advanced CM, SD-101 combined with pembrolizumab enhanced clinical efficacy by increasing local immune activation and improving T cell infiltration into the tumor, further supporting its role as an immunologic adjuvant [94].

PV-10, a 10% rose bengal disodium formulation, is a small molecule that exerts autolytic effects upon intralesional injection, leading to tumor cell necrosis and the subsequent activation of tumor-specific T cells. In a phase Ib clinical trial involving patients with advanced CM, the combination of PV-10 with pembrolizumab demonstrated favorable safety and early signs of clinical efficacy, with evidence of enhanced immune activation within injected lesions [95]. Building on these findings, PV-10 is currently being evaluated as a monotherapy in patients with various malignancies, including mUM (NCT00986661), to assess its potential to induce localized immune responses and systemic anti-tumor effects.

These agents represent a growing class of immune modulators capable of enhancing the therapeutic index of ICIs in tumors with low immunogenicity, such as mUM. Their integration into combination regimens may provide a more robust immune activation strategy, potentially overcoming resistance mechanisms that limit the efficacy of conventional checkpoint blockade.

#### 2.1.6. Melphalan and Regional Immunomodulation in mUM

Melphalan, a well-established alkylating chemotherapeutic agent, has traditionally been employed for its cytotoxic activity; however, accumulating evidence suggests that it also possesses immunomodulatory properties when administered regionally. In preclinical models, melphalan has been shown to induce local inflammatory responses that contribute to enhanced anti-tumor immunity. For example, in patients with limb-localized melanoma, ILP with melphalan significantly increased the local release of pro-inflammatory cytokines, including IL-1β, IL-6, and IL-8, thereby promoting immune cell recruitment and activation within the tumor microenvironment [21].

In mUM, melphalan is most often delivered through IHP or PHP, allowing for targeted drug delivery to hepatic metastases while limiting systemic toxicity. Clinical studies have demonstrated that IHP with melphalan significantly improves OS and PFS compared to conventional therapies, which may include systemic chemotherapy, immunotherapy, or other localized interventions. In a prospective trial, the 24-month OS rate was 46.5% in patients treated with melphalan-based IHP, compared to 29.5% in the control group receiving best alternative care [96]. Extended follow-up data further supported these findings, revealing a 3-year OS rate of 18.6% in the melphalan group versus 9.1% in controls and a 5-year OS rate of 16.3% compared to 6.8%. Median OS was also notably prolonged in the melphalan-treated cohort (21.4 months vs. 17.3 months) [97].

The potential to combine melphalan-induced regional immune activation with systemic immune checkpoint blockade has recently gained attention. In a phase Ib clinical trial evaluating IHP in conjunction with ICIs for the treatment of mUM, one arm of the study reported a high ORR of 57%, suggesting promising synergistic efficacy. However, this combination was also associated with significant adverse events, highlighting the need to optimize treatment protocols to improve tolerability while maintaining clinical benefit [98].

Taken together, these findings support the dual role of melphalan as both a cytotoxic and immunomodulatory agent. Its regional delivery via hepatic perfusion not only enhances direct tumor cell kill but may also modulate the immune microenvironment, laying the groundwork for combinatorial strategies with systemic immunotherapies in mUM.

#### 2.1.7. Targeting Immunosuppressive Pathways: IDO, TDO, and VEGF

In addition to classical immune checkpoints, several metabolic and angiogenic pathways contribute to immune suppression in the tumor microenvironment of UM, offering novel targets for therapeutic intervention. Among these, the enzymes indoleamine 2,3-dioxygenase (IDO) and tryptophan 2,3-dioxygenase (TDO), which mediate tryptophan catabolism, and the vascular endothelial growth factor (VEGF) axis play significant roles in immune evasion and tumor progression.

Due to its complex metabolic pathways and involvement in cancer progression, tryptophan metabolism is increasingly being studied as a therapeutic target in UM. IDO and TDO are key regulators of tryptophan metabolism, catalyzing the conversion of tryptophan into kynurenine and other downstream metabolites that suppress T cell and NK cell function and promote regulatory T cell expansion. The overexpression of these enzymes has been implicated in tumor-mediated immunosuppression across several cancer types [99,100]. In UM, elevated IDO expression has not been directly correlated with metastatic burden but is associated with increased immune cell infiltration and elevated levels of interferon-gamma (IFN-γ) within the tumor microenvironment [96]. Similarly, TDO has been found to be overexpressed in hepatic metastases of UM patients, suggesting a role in localized immune suppression and resistance to immune checkpoint inhibition [99]. The immunosuppressive effects of altered tryptophan metabolism may thus contribute to the poor responsiveness of UM to conventional immunotherapies, underscoring the need for further investigation into IDO and TDO as potential therapeutic targets [101]. Tryptophan is mainly converted into kynurenine, but it can also be metabolized in melanoma cells into serotonin and further into melatonin, a key hormone in controlling melanogenesis. As we mentioned earlier, this is another pathway that warrants high attention in future studies [102].

In parallel, VEGF, a critical mediator of angiogenesis, also exerts profound immunosuppressive effects by impairing dendritic cell maturation, promoting the recruitment of myeloid-derived suppressor cells (MDSCs), and inhibiting T cell trafficking into tumors. VEGF overexpression has been associated with increased tumor vascularization, metastatic potential, and poor clinical prognosis in multiple cancers, including melanoma [103]. A range of anti-VEGF therapeutics have received FDA approval for the treatment of multiple malignancies, including colorectal cancer, cervical cancer, non-small-cell lung cancer (NSCLC), renal cell carcinoma, and several other solid tumors [104]. In UM, VEGF-targeted therapies are currently under investigation for their potential to reverse VEGF-mediated immune suppression and enhance the anti-tumor immune response. Two humanized monoclonal antibodies against VEGF-A, ranibizumab and bevacizumab, are currently under clinical investigation for the treatment of mUM, either as monotherapy or in combination with other agents. Ongoing clinical trials include NCT00811200 and NCT00540930 for ranibizumab and NCT05075993, NCT02158520, NCT01471054, and NCT01217398 for bevacizumab.

These findings support a growing recognition that targeting immunosuppressive metabolic and angiogenic pathways may enhance the efficacy of immunotherapy in UM. The therapeutic inhibition of IDO, TDO, and VEGF—either alone or as part of combination strategies with ICIs or other immune-modulating agents—represents a promising avenue to overcome immune resistance and improve clinical outcomes in mUM.

#### 2.1.8. Cytokine-Based Approaches

Cytokine-based immunotherapy remains an important adjunct strategy in the treatment of mUM, particularly in combination with ICIs, peptide-based vaccines, and adoptive T cell therapies. Cytokines such as IL-2, interleukin-12 (IL-12), interferon-alpha (IFN-α), interferon-beta (IFN-β), and GM-CSF have demonstrated the ability to enhance the cytotoxic activity of both CD8+ T lymphocytes and NK cells while simultaneously inhibiting tumor cell proliferation.

In the context of UM, these cytokines are most commonly administered as part of combination regimens designed to amplify systemic immune responses. IL-2 and IFN-α have already received FDA approval for the treatment of several cancers, including metastatic CM [105,106], and their immunostimulatory properties continue to be evaluated in UM-specific clinical trials. Advancements in cytokine engineering have led to the development of more targeted immunotherapeutic agents with improved safety profiles. REGN10597, a receptor-masked IL-2 immunocytokine fused to a PD-1-targeting antibody, is currently undergoing evaluation in phase I/II clinical trials. Preclinical studies have shown that this molecule effectively controls tumor growth without eliciting the systemic toxicities traditionally associated with high-dose IL-2 therapy [107]. These findings highlight the promise of next-generation cytokine therapies to potentiate anti-tumor immunity while minimizing treatment-related adverse effects.

**Table 2:** Clinical trials investigating ICIs and other humanized mAb in patients with mUM, registered on ClinicalTrials.gov.

Treatment	Conditions	Phase	Actual Enrollment	Trial Period	Sponsor/Collaborators	Status	NCT No. (Reference)
REGN10597	Melanoma; Clear-Cell Renal-Cell Carcinoma (ccRCC); Advanced Solid Tumors	I/II	150	September 2024–March 2030	Regeneron Pharmaceuticals (Tarrytown, NY, USA)	Recruiting	NCT06413680
Ipilimumab + Nivolumab + PHP	UM: Liver metastases	III	40	June 2024–December 2030	Vastra Gotaland Region (Västra Götaland County, Sweden)	Recruiting	NCT06519266
Cemiplimab + Ziv-Aflibercept	mUM	II	32	February 2024–October 2030	H. Lee Moffitt Cancer Center and Research Institute (Tampa, FL, USA); Genzyme (Cambridge, MA, USA); Regeneron Pharmaceuticals (Tarrytown, NY, USA)	Recruiting	NCT06121180
Cemiplimab + ONM-501	UM; Multiple Cancers	I	168	October 2023–August 2026	OncoNano Medicine, Inc. (Dallas, TX, USA)	Recruiting	NCT06022029
Pembrolizumab + Olaparib	UM; Ocular Melanoma	II	37	October 2022–July 2026	H. Lee Moffitt Cancer Center and Research Institute (Tampa, FL, USA); Merck Sharp & Dohme LLC (Chalfont, PA, USA)	Recruiting	NCT05524935
Tislelizumab + Sitravatinib	UM	II	16	September 2022–May 2024	Grupo Español Multidisciplinar de Melanoma (Barcelona, Spain); Mirati Therapeutics Inc. ( San Diego, CA, USA); BeiGene (San Carlos, CA, USA)	Active, not recruiting	NCT05542342
Pembrolizumab + Lenvatinib	mUM	II	30	August 2022–June 2027	Providence Health & Services Merck Sharp & Dohme LLC (Chalfont, PA, USA); Eisai Inc (Exton, PA, USA).	Recruiting	NCT05308901
Pembrolizumab + Lenvatinib	mUM	II	54	July 2022–September 2028	Institut Curie, Merck Sharp & Dohme LLC (Chalfont, PA, USA)	Recruiting	NCT05282901
Relatlimab + Nivolumab + SBRT	mUM	II	40	September 2021–March 2026	California Pacific Medical Center Research Institute (San Francisco, CA, USA)	Recruiting	NCT05077280
LVGN3616 + LVGN6051 + LVGN7409 + Bevacizumab + Nab-Paclitaxel + Cyclophosphamide	mUM; Multiple Cancers	I	352	November 2021–February 2027	M.D. Anderson Cancer Center (Houston, TX, USA); Lyvgen Biopharma Holdings Limited (San Diego, CA, USA)	Active, not recruiting	NCT05075993
Ipilimumab + Nivolumab with Novacure Optune	mUM.	I	10	February 2022–August 2025	HonorHealth Research Institute, NovoCure Ltd. (Scottsdale, AZ, USA)	Recruiting	NCT05004025
Pembrolizumab + Dacetuzumab (SEA-CD40) + Pemetrexed + Carboplatin	Melanoma; Carcinoma, Non-Small-Cell Lung	II	77	October 2021–October 2025	Seagen Inc. (South San Francisco, CA, USA); Merck Sharp & Dohme LLC (Chalfont, PA, USA)	Active, not recruiting	NCT04993677
Ipilimumab/Relatlimab + Nivolumab + SD-101	mUM in the Liver	I	80	August 2021–January 2025	TriSalus Life Sciences, Inc. (Westminster, CO, USA)	Active, not recruiting	NCT04935229
Spartalizumab/ Tislelizumab	UM; Multiple Cancers	II	184	April 2021–March 2027	SOLTI Breast Cancer Research Group (Barcelona, Spain)	Recruiting	NCT04802876
Nivolumab + Relatlimab	mUM	II	27	November 2020–December 2026	Jose Lutzky, MD, University of Miami (Miami, FL, USA); Bristol-Myers Squibb (New York, NY, USA); United States Department of Defense	Active, not recruiting	NCT04552223
RO7293583 + Tocilizumab + Obinutuzumab + Adalimumab	CM; UM; Mucosal Melanoma	I	20	October 2020–July 2022	Hoffmann-La Roche (Basel, Switzerland)	Completed	NCT04551352
Ipilimumab + Nivolumab + IHP with melphalan	UM; Liver Metastases	I	18	March 2021–December 2024	Vastra Gotaland Region (Västra Götaland County, Sweden); Bristol-Myers Squibb (New York, NY, USA)	Active, not recruiting	NCT04463368
Avelumab + IOA-244 + Pemetrexed + Cisplatin + Ruxolitinib	Solid Tumor; Non-Hodgkin Lymphoma; NSCLC; Myelofibrosis; UM	I	210	February 2020–March 2025	iOnctura (Geneva, Switzerland)	Active, not recruiting	NCT04328844
Ipilimumab + Nivolumab + Melphalan CS-PHP	mUM	Ib/II	83	December 2019–December 2024	HW Kapiteijn, Leiden University Medical Center (Leiden, Netherlands)	Unknown	NCT04283890 [108,109]
Pembrolizumab + LNS8801	Solid Tumor (Adult)	I/II	200	October 2019–November 2024	Linnaeus Therapeutics, Inc. (Haddonfield, NJ, USA); Merck Sharp & Dohme LLC (Chalfont, PA, USA)	Recruiting	NCT04130516
Nivolumab + Pembrolizumab	mUM	NR	100	November 2016–December 2019	Institut Curie (Paris, France)	Unknown	NCT03964298
Nivolumab + Ipilimumab + Arginine deprivation (ADI-PEG 20)	UM	I	9	April 2019–January 2023	Memorial Sloan Kettering Cancer Center (New York, NY, USA)	Completed	NCT03922880
Nivolumab/Ipilimumab + IL-2 after radiation	Metastatic Melanoma	II	4	May 2019–December 2023	Masonic Cancer Center, University of Minnesota (Minneapolis, MN, USA)	Completed	NCT03850691
INCAGN02390	Melanoma; Multiple Cancers	I	40	September 2018–August 2021	Incyte Corporation (Wilmington, DE, USA)	Completed	NCT03652077
Nivolumab + Ipilimumab	Melanoma, Ocular Melanoma	II	52	July 2018–June 2023	Suthee Rapisuwon (Washington, DC, USA); Bristol-Myers Squibb (New York, NY, USA)	Active, not recruiting	NCT03528408
Pembrolizumab + APG-115	UM; Multiple Cancers	I/II	230	August 2018–March 2025	Ascentage Pharma Group Inc. (Rockville, MD, USA); Merck Sharp & Dohme LLC (Chalfont, PA, USA)	Recruiting	NCT03611868
Ipilimumab + Nivolumab + Immuno-embolization	Metastatic Malignant Neoplasm in the Liver; mUM; Stage IV UM	II	14	May 2018–December 2024	Sidney Kimmel Cancer Center at Thomas Jefferson University (Philadelphia, PA, USA); Bristol-Myers Squibb (New York, NY, USA)	Active, not recruiting	NCT03472586
Nivolumab	mUM; Multiple Cancers	I	70	May 2018–December 2025	M.D. Anderson Cancer Center (Houston, TX, USA); NCI (Bethesda, MD, USA)	Recruiting	NCT03025256
Ipilimumab + Nivolumab + SIR-Spheres Yttrium90	UM; Liver Metastases	I/II	26	October 2016–June 2023	David Minor, MD; California Pacific Medical Center (San Francisco, CA, USA); Jefferson Medical College of Thomas Jefferson University (Philadelphia, PA, USA); University of Chicago (Chicago, IL, USA)	Unknown	NCT02913417
Pembrolizumab + SRS	Ocular Melanoma; Multiple Cancers	I	27	October 2016–October 2023	Emory University (Atlanta, GA, USA); Merck Sharp & Dohme Corp. (Chalfont, PA, USA)	Completed	NCT02858869
ICON-1	UM; Choroid Neoplasm	I	10	May 2016–September 2017	Iconic Therapeutics, Inc. (San Francisco, CA, USA)	Completed	NCT02771340
Pembrolizumab + Entinostat	mUM	II	29	February 2018–January 2023	Vastra Gotaland Region (Västra Götaland County, Sweden); Merck Sharp & Dohme Corp. (Chalfont, PA, USA); Syndax Pharmaceuticals (Waltham, MA, USA)	Completed	NCT02697630
Nivolumab + Ipilimumab	UM	II	52	April 2016–July 2021	Grupo Español Multidisciplinar de Melanoma (Barcelona, Spain); Bristol-Myers Squibb (New York, NY, USA)	Completed	NCT02626962
Ipilimumab + Nivolumab + Relatlimab	CM; UM; Mucosal Melanoma; Ocular Melanoma; Acral Lentiginous Melanoma	II	53	February 2016–January 2023	M.D. Anderson Cancer Center (Houston, TX, USA); NCI (Bethesda, MD, USA)	Completed	NCT02519322 [91]
Pembrolizumab	UM (stage III-IV)	II	5	May 2015–August 2019	Vanderbilt-Ingram Cancer Center (Nashville, TN, USA); NCI (Bethesda, MD, USA)	Terminated	NCT02359851
Ipilimumab + Bevacizumab + Nab-paclitaxel	Metastatic Melanoma; Mucosal Melanoma; CM; UM; Unresectable Melanoma	II	24	October 2013–October 2019	Academic and Community Cancer Research United (SWRochester, MN, USA); NCI (Bethesda, MD, USA)	Completed	NCT02158520
Ipilimumab + SIR-Spheres Yttrium90	Ocular and extraocular melanoma; Liver Metastases	I	6	December 2012–February 2016	Case Comprehensive Cancer Center (Cleveland, OH, USA); NCI (Bethesda, MD, USA)	Terminated (Research canceled)	NCT01730157
Nivolumab + Ipilimumab	mUM; Stage IV UM	II	67	November 2012–May 2024	MD Anderson Cancer Center (Houston, TX, USA); NCI (Bethesda, MD, USA)	Completed	NCT01585194
INF-α-2b + Dacarbazine	Ciliary Body and Choroid Melanoma; Iris Melanoma; Recurrent Intraocular Melanoma	II	38	November 2009–December 2017	Case Comprehensive Cancer Center (Cleveland, OH, USA)	Completed	NCT01100528
Tremelimumab (CP-675,206)	UM	II	11	August 2009–August 2017	AHS Cancer Control Alberta (Alberta, Canada)	Completed	NCT01034787
PV-10	mUM; Multiple Cancers	I	78	October 2009–February 2023	Provectus Biopharmaceuticals, Inc. (Knoxville, TN, USA)	Unknown	NCT00986661
GM-CSF by embolization	UM; Liver Metastases	II	53	October 2004–June 2012	Sidney Kimmel Cancer Center at Thomas Jefferson University (Philadelphia, PA, USA); NCI	Completed	NCT00661622
Radiolabeled monoclonal antibody: iodine I 131 mAb 3F8	Intraocular Melanoma; Multiple cancers	II	78	January 2006–February 2023	Memorial Sloan Kettering Cancer Center (New York, NY, USA)	Completed	NCT00445965
Pegylated INF-α-2b + Thalidomide	Intraocular Melanoma; Melanoma (Skin)	II	32	January 2001–June 2007	Barbara Ann Karmanos Cancer Institute; NCI (Bethesda, MD, USA)	Completed	NCT00238329
INF-β	Stage IV Melanoma; Recurrent Melanoma	II	21	April 2004–October 2007	Case Comprehensive Cancer Center (Cleveland, OH, USA); NCI (Bethesda, MD, USA)	Completed	NCT00085306
Ipilimumab + IL-2	Intraocular Melanoma; Melanoma (Skin)	I/II	Not mentioned	February 2003–August 2006	NCI (Bethesda, MD, USA)	Completed	NCT00058279
Pegylated INF-α + Temozolomide	Intraocular Melanoma; Melanoma (Skin)	II	Not mentioned	May 2001–June 2005	Memorial Sloan Kettering Cancer Center (New York, NY, USA); NCI (Bethesda, MD, USA)	Completed	NCT00027742

CM: cutaneous melanoma; CS-PHP: chemosaturation via percutaneous hepatic perfusion; GM-CSF: granulocyte-macrophage colony stimulating factor; ICON-1: human immuno-conjugate 1; IHP: isolated hepatic perfusion; IL-2: interleukin 2; INF: interferon; mAb: monoclonal antibody; mUM: metastatic uveal melanoma; NCI: National Cancer Institute; NR: not reported: NSCLC: non-small-cell lung cancer; SBRT: stereotactic body radiotherapy; SRS: stereotactic radiosurgery; UM: uveal melanoma; PHP: percutaneous hepatic perfusion.

As understanding of the tumor immune microenvironment in UM continues to evolve, cytokine-based interventions are likely to remain an essential component of integrated immunotherapeutic strategies, particularly in overcoming immune resistance and enhancing the efficacy of existing modalities.

Despite the transformative success of ICIs in other malignancies, their benefit in mUM remains limited due to intrinsic immunoresistance and a uniquely suppressive tumor microenvironment. Combination strategies—including dual checkpoint blockade, integration with locoregional therapies, and novel immune targets—have yielded improved response rates but are often constrained by toxicity and heterogeneity in response. The continued clinical evaluation of emerging targets such as TIM-3, LAG-3, and TIGIT, along with adjunctive use of cytokines and immune adjuvants, holds promise for overcoming resistance mechanisms in mUM. The pursuit of rationally designed multimodal immunotherapeutic strategies tailored to the molecular and immune context of mUM is essential for advancing patient outcomes.

### 2.2. T Cell Receptor (TCR)-Based Therapies in Patients with mUM

TCR-based immunotherapy represents a promising and rapidly advancing strategy for the treatment of mUM. By engineering or mobilizing T cells to specifically recognize tumor-associated antigens, TCR-based therapies aim to generate robust, tumor-directed cytotoxic responses. These therapies can be classified into two primary categories: non-cellular TCR-based therapies, which involve synthetic molecules such as bispecific TCRs, and cellular TCR-based therapies, which involve the adoptive transfer of autologous or genetically modified T cells.

#### 2.2.1. Non-Cellular TCR-Based Therapy

Among non-cellular TCR-based immunotherapies, bispecific TCR antibodies have emerged as potent agents capable of redirecting endogenous T cells toward tumor cells by concurrently engaging a tumor-specific antigen and the CD3 receptor on T cells. These bispecific constructs represent a novel class of therapeutics that offer promising alternatives to conventionally targeted anti-cancer therapies. They are specifically engineered to activate CD3+ T cells in the presence of tumor-associated antigens, immune checkpoint ligands, cytokines, or key signaling molecules. Several bispecific antibodies have already received FDA approval for the treatment of various malignancies, including hematologic cancers (e.g., blinatumomab for B-cell acute lymphoblastic leukemia), metastatic non-small-cell lung cancer (e.g., amivantamab), and mUM (e.g., tebentafusp). Numerous additional bispecific constructs are currently under evaluation in clinical trials across a broad spectrum of tumor types [111].

Tebentafusp (IMCgp100) is the first bispecific TCR-based agent approved for the treatment of mUM. It belongs to the class of immune-mobilizing monoclonal TCRs against cancer (ImmTACs) and consists of a soluble, high-affinity TCR domain that specifically recognizes the HLA-A*02:01-restricted gp100 peptide, an antigen widely expressed in UM, fused to an anti-CD3 single-chain variable fragment (scFv) designed to recruit and activate polyclonal CD3+ T cells [112,113]. Upon engagement with gp100-expressing tumor cells, tebentafusp facilitates the recruitment of both CD4+ and CD8+ T lymphocytes, resulting in T cell activation, pro-inflammatory cytokine production, and tumor cell lysis. Clinical studies have demonstrated that treatment with tebentafusp results in significant increases in circulating levels of CXCL10, CXCL11, IL-2, IL-6, and IL-10, as well as a reduction in CXCR3+CD8+ T cells in peripheral blood [78]. These findings suggest enhanced T cell infiltration into the tumor microenvironment and the effective redirection of effector T cells toward tumor sites.

Tebentafusp received FDA approval in January 2022 following the pivotal phase III IMCgp100-202 trial, which demonstrated significantly improved survival outcomes [18]. In this trial, 252 HLA-A*02:01-positive mUM patients were randomized to receive tebentafusp or investigator’s choice of therapy (ICIs or chemotherapy). The tebentafusp group achieved a 1-year OS rate of 73% compared to 59% in the control group, and 6-month PFS was 31% versus 19%, respectively [18]. With a minimum follow-up of 36 months, the median OS was 21.6 months in the tebentafusp group versus 16.9 months in the control group, and 3-year OS rates were 27% versus 18%, respectively [114].

Tebentafusp has also shown favorable tolerability. The majority of adverse events were mild to moderate and primarily cutaneous or cytokine-mediated, in contrast to the more severe immune-related toxicities seen with ICIs, such as colitis, pneumonitis, and endocrinopathies [18,80,82]. Real-world data from a French cohort further validated these findings, where 33 of 60 treated patients achieved stable disease (SD) and five had partial responses (PR), with a median PFS of 28 weeks and a 1-year OS rate of 74%, closely mirroring outcomes from the phase III trial [115].

Ongoing clinical trials are currently evaluating tebentafusp in combination with ICIs to explore potential synergy (Table 3). Additionally, computational modeling using a quantitative systems pharmacology (QSP) approach has identified key predictors of response, including elevated intratumoral CD8+ T cell density, increased CD4+ helper T cell infiltration, and a higher CD8+/Treg ratio in responders versus non-responders, offering potential biomarkers for patient stratification [116].

#### 2.2.2. Cellular TCR-Based Therapy

Cellular TCR-based immunotherapy is an evolving therapeutic strategy in tumors that involves the ex vivo expansion or the genetic engineering of autologous T cells to enhance their antigen specificity and cytolytic activity against tumor cells [106]. This strategy includes both non-engineered tumor-infiltrating lymphocyte (TIL) therapy and genetically modified TCR-T cell approaches targeting defined melanoma-associated antigens.

Early clinical efforts using unmodified TILs have demonstrated feasibility and modest clinical benefit in mUM. In one study involving 96 patients with UM, TILs were successfully harvested and expanded from patient tumors, and nine patients subsequently received TIL therapy. Among these, 22% achieved PR with durations ranging from 16.5 to 22.1 months, 44% experienced SD, and 33% had PD, supporting the potential of TIL-based approaches [117]. A separate phase I clinical trial (NCT01814046) evaluating adoptive TIL transfer in 21 patients with mUM reported objective tumor regression in 35% of cases, with 29% achieving PR and one patient demonstrating a CR [10]. However, all patients experienced significant immune-related toxicities [10], highlighting the challenges of balancing efficacy with tolerability. Several clinical trials remain ongoing to evaluate autologous TILs, either as monotherapy or in combination with immune-modulatory agents.

Genetically modified T cell therapies have provided a more targeted approach to cancer immunotherapy by equipping patient-derived T cells with TCRs specific to tumor-associated antigens. Two main platforms exist: TCR-engineered T cells (TCR-T) and chimeric antigen receptor T cells (CAR-T). While CAR-T cells are advantageous in hematologic malignancies due to their MHC-independent recognition of surface antigens, they are less effective in solid tumors such as mUM. In contrast, TCR-T cells can recognize intracellular peptides presented via HLA molecules and can engage a broader repertoire of tumor antigens with higher sensitivity, albeit restricted to patients with specific HLA alleles such as HLA-A*0201 [118,119,120].

One example of CAR-T therapy investigated in UM is the use of C7R-GD2-specific CAR-T cells in a phase I trial (NCT03635632), which recruited patients with UM and other GD2-expressing solid tumors. GD2, a disialoganglioside overexpressed in tumors such as neuroblastoma, small-cell lung cancer, and melanoma, is a compelling target due to its association with high tumor invasiveness and proliferation. Anti-GD2 monoclonal antibodies are already in clinical use for pediatric neuroblastoma, and GD2-specific CD8+ T cells represent a promising strategy for immunotherapy in GD2-positive tumors [119].

TCR-T therapies have been developed to target a range of melanoma-associated antigens expressed in UM, including PRAME (preferentially expressed antigen in melanoma), MAGE-C2 (melanoma-associated antigen C2), SLC45A2, and MART-1 (melanoma antigen recognized by T cells-1). Approximately 50% of primary and metastatic UM tumors express PRAME [121,122], and T cells engineered with PRAME-specific TCRs have demonstrated selective cytotoxicity against PRAME-positive melanoma cell lines [123]. Similarly, MAGE-C2, expressed in 39% of melanomas and absent in normal tissues, and SLC45A2, a melanocyte lineage antigen highly expressed in UM cell lines but minimally in normal tissues, have also been validated as effective TCR targets. SLC45A2-specific CD8+ T cells showed robust cytolytic activity against HLA-matched mUM cells [122,124]. Additionally, MART-1, a melanoma antigen recognized by T cells and commonly expressed in UM, has been a focus of multiple immunotherapeutic studies, with genetically modified T cells capable of selectively eliminating MART-1-positive melanoma cells [112,125].

Several TCR-T cell therapies targeting these antigens have reached clinical evaluation in mUM (Table 3). PRAME-specific TCR-T cells have been investigated in the NCT02743611 trial for patients with advanced solid tumors. Among evaluable participants, approximately half of them experienced PR, and notably, one of two mUM patients in this study had a PR while the other achieved SD [11]. A separate phase Ib trial of SLC45A2-specific TCR-T therapy (NCT03068624) reported SD in 36% of treated patients, with an mPFS of 5.9 weeks and a median overall survival (mOS) of 8.9 weeks. Among patients with SD, the median duration of response was 5.7 months [126]. Unfortunately, MART-1-specific TCR-T therapy (NCT02654821), while biologically promising, demonstrated high toxicity and limited efficacy in a small cohort of 12 melanoma patients, including UM [12]. Other trials targeting MAGE-C2 (NCT04729543) and SLC45A2 are ongoing, with further results awaited to determine safety and therapeutic potential.

Cellular TCR-based therapies offer a compelling strategy for mUM treatment by leveraging tumor-specific antigen recognition and T cell-mediated cytotoxicity. TILs represent a feasible, albeit toxic, approach with encouraging early efficacy, while genetically engineered TCR-T cells targeting PRAME, MART-1, SLC45A2, and other UM-associated antigens provide a more tailored strategy. Future directions will focus on improving antigen selection, reducing on-target off-tumor toxicity, optimizing HLA-restriction barriers, and developing combination regimens to potentiate TCR-T cell efficacy in the immunologically distinct microenvironment of UM.

## 3. Vaccine Therapy in Patients with mUM

Vaccine-based immunotherapy represents a promising and increasingly explored strategy in the treatment of mUM. Designed to prime and activate tumor-specific immune responses, cancer vaccines aim to stimulate cytotoxic T lymphocyte activity and promote durable antitumor immunity. Various vaccine platforms have been developed, including peptide-based vaccines (comprising tumor-associated antigens and adjuvants), nucleic acid-based vaccines (DNA or mRNA encoding tumor antigens), dendritic cell (DC)-based vaccines (autologous DCs pulsed with tumor-derived peptides or RNA), and viral vector-based vaccines (attenuated or oncolytic viruses carrying genes encoding tumor antigens) [127].

Although vaccine-based therapies have shown significant clinical benefits in certain cancers, such as the FDA-approved virus-like particle vaccines targeting human papillomavirus (HPV) in cervical cancer and hepatitis B virus in hepatocellular carcinoma [128], no cancer vaccine is currently approved for UM. Nevertheless, several experimental vaccine approaches are under active clinical investigation in mUM and show promising early signals of efficacy.

### 3.1. Cell-Based Vaccines in UM

Among the most studied vaccine modalities in mUM are cell-based vaccines employing autologous monocyte-derived DCs. These DCs are isolated from the patient, pulsed with tumor-associated antigens such as gp100 or tyrosinase peptides, or transfected with tumor-derived RNA through electroporation, and subsequently reinfused to induce a tumor-specific T cell response. In a pilot clinical study, DCs loaded with gp100 and tyrosinase peptides were administered to patients with mUM. This treatment was well tolerated and resulted in a mOS of 19.2 months, suggesting clinical benefit with minimal toxicity [129].

Combining cancer vaccines with ICIs may further enhance their immunogenic potential. This combinatorial strategy is based on the rationale that vaccines can increase tumor antigen presentation and T cell priming, while ICIs sustain effector T cell function by blocking inhibitory signaling pathways. Preliminary data from a small cohort of five mUM patients treated with a DC vaccine in combination with the PD-1 inhibitor pembrolizumab demonstrated remarkable outcomes [14]. The median OS reached 36.4 months, with no reported severe immune-related adverse events, and two patients achieved prolonged disease-free survival [14]. This represents one of the most promising clinical responses observed to date in mUM and underscores the therapeutic synergy between vaccines and ICIs (Figure 2D).

In addition to peptide-loaded DCs, other innovative vaccine strategies are being tested in clinical trials. One ongoing study (NCT04335890) is evaluating a DC vaccine loaded with tumor-derived RNA encoding for IκB kinase β (IKKβ), a key regulator of the NF-κB signaling pathway, known to modulate inflammation and immune responses in cancer. This vaccine aims to promote the activation of both CD4+ and CD8+ T cells, as well as natural killer (NK) cells, thereby orchestrating a multifaceted immune attack against UM [13].

### 3.2. Peptide-Based Vaccines in UM

#### 3.2.1. Tumor-Associated Antigens and Peptide Vaccine Design

Peptide-based cancer vaccines represent a rational immunotherapeutic approach for UM, aiming to stimulate tumor-specific cytotoxic T lymphocyte responses against defined tumor-associated antigens (TAAs). Several melanoma-specific peptides, including gp100, MART-1, MAGE proteins, and tyrosinase, have been widely studied as immunogenic targets in vaccine development. These peptides are derived from melanoma-specific proteins involved in pigmentation and melanocytic differentiation, particularly tyrosinase, an enzyme essential in melanin biosynthesis (Table 4). Upon administration, these peptides are processed and presented by antigen-presenting cells, especially DCs, through MHC class I and II molecules to prime CD8+ and CD4+ T cells. This mechanism initiates a tumor-specific adaptive immune response, which may promote tumor clearance or delay disease progression.

#### 3.2.2. Immunoadjuvants and Immune Potentiation Strategies

To enhance immunogenicity and overcome the typically low immunostimulatory potential of short peptides, peptide-based vaccines are commonly administered with potent immunological adjuvants. These include incomplete Freund’s adjuvant (IFA), alum, and synthetic CpG oligonucleotides, which promote a pro-inflammatory environment conducive to T cell priming. Additionally, cytokine co-administration such as IL-2 and IL-12, or GM-CSF, has been used to augment T cell proliferation and enhance DC recruitment and maturation [130]. Together, these combinations aim to facilitate the development of effective antitumor immunity by improving antigen presentation and enhancing T cell activation within the tumor microenvironment.

#### 3.2.3. Clinical Trials of Peptide-Based Vaccines in UM

Several clinical trials have explored peptide-based vaccines in UM patients, though many remain unpublished or in early developmental stages (Table 4). A notable precedent is the gp100 peptide vaccine combined with high-dose IL-2, evaluated in a randomized phase III trial (NCT00019682) in patients with advanced melanoma. This study demonstrated a significant improvement in objective response rate and progression-free survival (PFS), setting a clinical benchmark for peptide vaccines in melanoma [131]. However, this trial did not specifically focus on UM, underscoring the need for subtype-specific immunotherapeutic evaluation.

In UM, a recent clinical trial (NCT04364230) investigated a combinatorial peptide vaccine approach incorporating six melanoma helper peptides (6MHP) and a mutated BRAF-derived peptide (NeoAg-mBRAF), co-formulated with immune adjuvants PolyICLC (a Toll-like receptor 3 agonist) and CDX-1140 (a CD40 agonist). These adjuvants were selected to potentiate T-helper type 1 (Th1) responses and promote effective cytotoxic T lymphocyte activation. Preliminary findings suggest that the 6MHP-based vaccine, when combined with IFA, PolyICLC, and/or low-dose metronomic cyclophosphamide (mCy), elicited robust T cell responses and was well-tolerated in melanoma patients, including those with UM [132].

A retrospective abstract review of polypeptide vaccine trials conducted at the University of Virginia between 2012 and 2022 provided additional insight into the clinical feasibility and immunogenicity of these vaccines in UM [133]. The cohort included 11 UM patients, 10 of whom received the 6MHP vaccine and one who received the MELITAC 12-peptide vaccine. Of these, two patients discontinued participation due to adverse effects, whereas among the remaining nine patients, five demonstrated peptide-specific immune responses. Although statistical significance was not reached, these patients exhibited improved relapse-free survival (RFS) and OS. Notably, patients who mounted a measurable T cell response showed a 100% three-year RFS and OS rate, compared to 50% in non-responders, suggesting a potential association between vaccine-induced immune activation and long-term disease control [133].

### 3.3. Nucleic Acid-Based Vaccines in UM

#### 3.3.1. Mechanism and Rationale for Nucleic Acid-Based Vaccines

Nucleic acid-based vaccines, encompassing both DNA and RNA platforms, represent an innovative class of cancer immunotherapies designed to elicit targeted antitumor immune responses through in vivo expression of TAAs. These vaccines deliver genetic material—either plasmid DNA or messenger RNA (mRNA)—encoding specific tumor antigens, sometimes alongside immunostimulatory molecules such as cytokines (e.g., IL-2, IL-12, GM-CSF), to promote robust and durable immune activation. Once internalized by host antigen-presenting cells, particularly DCs, the encoded antigens are expressed and processed for presentation via both MHC class I and II pathways, thereby initiating cytotoxic CD8+ T cell responses and CD4+ helper T cell-mediated immunity.

DNA vaccines, typically delivered via bacterial plasmids, can be administered with recombinant protein adjuvants or engineered to co-express cytokines that enhance T cell priming. RNA vaccines, which are inherently more labile, often require formulation with protective carriers such as protamine complexes or lipid-based nanoparticles (liposomes) to prevent degradation and facilitate cellular uptake. Both platforms aim to induce a systemic immune response capable of controlling tumor growth and preventing metastatic spread. Although these vaccines are generally well tolerated and safe, clinical trials have frequently reported suboptimal immunogenicity in melanoma patients, highlighting the need for improved delivery systems and vaccine constructs [134,135].

#### 3.3.2. Clinical Evidence and Application in UM and CM

To date, there are no active clinical trials specifically evaluating nucleic acid-based vaccines exclusively in patients with UM. However, limited data from completed trials involving mixed cohorts of melanoma patients, including UM cases, provide preliminary insights. Two clinical studies registered on ClinicalTrials.gov evaluated chimeric DNA vaccines encoding xenogeneic tumor antigens such as mouse tyrosinase and gp100, leveraging the concept of breaking tolerance to self-antigens with high sequence homology to human proteins (Table 4). These vaccines aim to generate cross-reactive T cell responses targeting conserved melanoma antigens.

In one trial (NCT00471133), a xenogeneic tyrosinase-encoding DNA vaccine was administered via electroporation, a technique that enhances plasmid uptake by host cells. The vaccine was well tolerated, and 62.5% of melanoma patients demonstrated an increase in anti-tyrosinase CD8+ T cell responses [12]. Another trial (NCT00398073) evaluated a mouse gp100 DNA vaccine delivered using particle-mediated epidermal delivery (PMED), which involves biolistic administration directly into the skin. This method induced an increase in IFNγ-producing CD8+ T cells and enhanced memory responses in approximately 30% of patients. Compared to conventional intramuscular delivery, PMED appeared superior, likely due to its enhanced stimulation of innate immunity and local inflammatory signals [16,136].

Messenger RNA-based vaccines have also been investigated in CM with emerging success. Both unmodified (“naked”) mRNA and protamine-complexed mRNA formulations have demonstrated safety and moderate immunogenicity in early-phase studies, with antigen-specific immune responses observed in subsets of patients [137,138]. However, limited antigen expression duration and the innate immune sensing of RNA components may restrict the amplitude of the immune response, necessitating optimized formulations.

Recently, a novel liposomal mRNA vaccine platform, referred to as FixVac, was tested in patients with unresectable melanoma. FixVac encoded four non-mutated melanoma-associated antigens: NY-ESO-1, MAGE-A3, tyrosinase, and TPTE. When administered alone or in combination with PD-1 blockade, this vaccine elicited strong tumor-specific CD4+ and CD8+ T cell responses, reinforcing the therapeutic potential of RNA vaccines in melanoma immunotherapy [139]. Although UM was not the primary focus of this study, the antigens used (e.g., tyrosinase and MAGE-A3) are expressed in UM as well, suggesting potential cross-applicability.

### 3.4. Viral-Based Vaccines in UM

#### 3.4.1. Mechanism and Rationale of Viral-Based Vaccines

Viral-based vaccines utilize engineered viral vectors to deliver TAAs directly into antigen-presenting cells (APCs), thereby stimulating both innate and adaptive immune responses. Unlike peptide or nucleic acid vaccines, viral vectors, such as modified vaccinia Ankara (MVA), vaccinia virus (VV), alphaviruses, and avipoxviruses, possess intrinsic immunostimulatory properties, often eliminating the need for external adjuvants [140]. Upon infection of host APCs, these vectors express the encoded TAAs endogenously, leading to presentation via MHC class I molecules and robust activation of cytotoxic CD8+ T lymphocytes against tumor cells. This mechanism of action makes viral-based vaccines particularly attractive for eliciting strong systemic immune responses in melanoma, including UM.

#### 3.4.2. FDA-Approved and Investigational Viral Platforms in Melanoma

Talimogene laherparepvec (T-VEC) represents a landmark example of an oncolytic viral vaccine approved for clinical use. T-VEC is a genetically modified herpes simplex virus type 1 (HSV-1) engineered to express GM-CSF. Administered via direct intratumoral injection, T-VEC induces the local viral lysis of tumor cells and GM-CSF-mediated recruitment of DCs, thereby enhancing both local and systemic antitumor immunity. The U.S. FDA approved T-VEC for the treatment of unresectable CM, although its application in UM remains investigational [141].

Another promising vector-based immunotherapy is Coxsackievirus A21 (CVA21), an oncolytic RNA virus with preferential tropism for ICAM-1-expressing tumor cells. In a phase I clinical trial (NCT02307149), CVA21 combined with ipilimumab demonstrated an ORR of 30%, median progression-free survival (mPFS) of 6.2 months, and mOS of 45.1 months in melanoma patients, with an acceptable safety profile [142]. Although these results are encouraging, their translation to UM is still under investigation. CVA21 was subsequently tested in a cohort of 11 patients with mUM (NCT03408587), but the study has not yet reported outcomes.

#### 3.4.3. Active Clinical Development of Viral Vaccines in UM

Currently, an active clinical trial (NCT03865212) is evaluating a recombinant vesicular stomatitis virus (VSV) platform expressing IFNβ and tyrosinase-related protein 1 (TYRP1), a melanoma-specific antigen. This novel viral construct, VSV-IFNβ-TYRP1, is designed to simultaneously promote direct tumor cytotoxicity and immune activation. The trial includes both CM and mUM patients, and the outcomes are anticipated to inform the potential of this approach for uveal-specific immunotherapy.

One of the most advanced viral immunotherapeutics currently being evaluated for UM is RP2, a next-generation oncolytic HSV-1 vector. RP2 has been genetically modified to enhance oncolytic activity and immunogenicity by incorporating GM-CSF, a deletion of the R sequence from the gibbon ape leukemia virus (GALV-GP R-), and the expression of an anti-CTLA-4 antibody-like molecule. These modifications are intended to promote tumor-selective replication, enhance immune cell recruitment, and provide a checkpoint blockade to overcome T cell exhaustion. A phase I clinical trial evaluating RP2 monotherapy or in combination with nivolumab enrolled 17 patients with mUM, of whom 14 received combination therapy. The ORR was 29.4%, and the DCR reached 58.8% [143]. Notably, patients with clinical benefit exhibited increased the intratumoral CD8+ T cell infiltration and upregulation of PD-L1 expression, suggesting the immune remodeling of the tumor microenvironment [143]. A larger phase II/III trial (NCT06581406) is now recruiting to further assess efficacy and safety in mUM patients.

### 3.5. Summary and Future Perspectives

Vaccine-based immunotherapy is an emerging approach in mUM, aiming to harness TAAs to prime effective antitumor T cell responses. Although no vaccine strategy has yet been approved for mUM, multiple platforms, including DC-based, peptide-based, nucleic acid-based, and viral vector-based vaccines, are under active investigation. Early-phase studies have demonstrated favorable safety and immunogenicity, with growing evidence that combination strategies, particularly with ICIs, may enhance therapeutic efficacy.

Peptide-based vaccines targeting melanoma antigens such as gp100, MART-1, and tyrosinase have shown preliminary immune activation, especially when paired with adjuvants or cytokines that augment antigen presentation. However, most studies remain limited in scale and lack UM-specific outcome data. The development of neoantigen-based vaccines and integration with immune checkpoint blockade may enhance both specificity and clinical benefit.

Nucleic acid-based vaccines offer scalability and customization but have shown limited immunogenicity in early trials. Advances such as liposomal mRNA platforms (e.g., FixVac) have improved immune activation in melanoma and hold promise for UM when adapted to its unique antigenic and immunologic landscape. Tailored delivery strategies and refined antigen selection will be critical for success in this context.

Viral vector-based vaccines, including oncolytic viruses like T-VEC, CVA21, and RP2, have demonstrated the ability to elicit robust systemic immune responses by combining direct tumor lysis with immune stimulation. Early data in mUM, particularly with RP2 in combination with nivolumab, are encouraging. However, further validation in UM-specific cohorts is needed, especially considering the distinct immune microenvironment of the eye and liver.

Overall, vaccine therapy has the potential to become a key component of multimodal immunotherapy in mUM. The continued optimization of vaccine platforms, rational combinations with immune modulators, and biomarker-driven patient selection will be essential to fully realize their therapeutic potential in this challenging melanoma subtype.

## 4. Discussion

mUM remains a highly lethal malignancy with limited therapeutic options and consistently poor prognosis. Unlike CM, mUM exhibits a low tumor mutational burden, a distinct immune microenvironment, and a strong predilection for hepatic metastasis—factors that collectively contribute to resistance against conventional immunotherapies. Despite the transformative impact of ICIs in many solid tumors, the majority of mUM patients derive minimal benefit from ICI monotherapy, underscoring the urgent need for alternative or combinatorial approaches. Ongoing clinical trials exploring novel immunotherapeutics for mUM carry significant promise and represent a critical step forward in addressing this unmet clinical need.

The use of dual immune checkpoint inhibition, particularly the combination of anti-CTLA-4 (ipilimumab) with anti-PD-1 agents (nivolumab or pembrolizumab), has shown improved efficacy over monotherapy in mUM, as reflected in modestly higher response rates and longer progression-free survival in certain patient subsets. However, its clinical utility remains constrained by two significant challenges: limited therapeutic benefit and frequent occurrence of high-grade immune-related adverse events (irAEs). These toxicities, which commonly affect multiple organ systems, including the gastrointestinal tract (colitis), liver (hepatitis), endocrine system (thyroiditis, hypophysitis), musculoskeletal tissue (myositis), and skin (dermatitis), often compromise treatment tolerability and patient outcomes.

The critical analysis of clinical data from 194 mUM patients revealed that 44.7% experienced grade 1–2 toxicities, while 29.4% developed grade 3–4 adverse events. Interestingly, patients with severe irAEs demonstrated significantly longer overall survival (29 months versus 14.5 months in those with no or mild toxicity), suggesting that immune-related toxicity may serve as a potential biomarker for treatment response and prognostic evaluation [144]. This paradoxical association between irAE severity and survival benefits further investigation to elucidate the underlying immunological mechanisms. The variability in reported toxicity profiles across studies stems from multiple factors, including heterogeneous patient populations, differences in therapeutic protocols, and limited sample sizes in most clinical trials. These observations underscore the importance of optimizing the risk-benefit ratio through careful patient selection, individualized dosing strategies, and proactive management of immune-mediated toxicities. Future research should focus on identifying predictive biomarkers to better stratify patients who are most likely to benefit from this aggressive immunotherapeutic approach while minimizing unnecessary toxicity.

A major breakthrough in the immunotherapeutic landscape for mUM came with the recent FDA approval of tebentafusp (IMCgp100), a bispecific T cell engager that targets gp100 presented by HLA-A02:01 and recruits polyclonal T cells to kill melanoma cells. Tebentafusp has demonstrated a significant overall survival benefit in HLA-A02:01–positive patients with previously untreated mUM. Notably, its safety profile proves markedly superior to conventional immune checkpoint inhibitors (ICIs), with no treatment-related fatalities reported and primarily grade 1–2 adverse events limited to cutaneous reactions (rash, pruritus) and transient cytokine-mediated symptoms (fever, fatigue) [18,80]. Ongoing clinical investigations are evaluating tebentafusp in combination with ICIs or in sequential regimens, with the goal of further enhancing therapeutic efficacy while preserving tolerability. For a large number of patients ineligible for tebentafusp therapy (primarily HLA-A*02:01-negative individuals), there remains an urgent need to develop alternative targeted therapies.

Beyond ICIs and bispecific T cell engagers, cell-based immunotherapies and therapeutic vaccines are being actively explored as potential avenues to overcome the immune resistance characteristic of mUM. Early-phase trials involving adoptive T cell therapies, TILs, and genetically engineered TCR therapies have shown preliminary signs of efficacy, albeit in small and heterogeneous cohorts. The challenge remains in optimizing T cell persistence, trafficking to hepatic metastases, and avoiding off-target toxicities.

Vaccine-based immunotherapies—whether peptide-based, nucleic acid-based, DC-based, or viral vector-based—are increasingly gaining attention for their ability to induce tumor-specific immune responses with relatively low toxicity. While most of these approaches are still in early development or under clinical evaluation, preliminary data suggest that vaccines may enhance antigen-specific T cell priming, especially when combined with immune-modulating agents such as ICIs or cytokines. Tailoring these platforms to the unique antigenic and immunological profile of mUM will be essential for maximizing their therapeutic potential.

Despite the challenges inherent to treating mUM, recent progress in immunotherapy—particularly through tebentafusp and evolving vaccine and cell-based strategies—has introduced new optimism into a historically refractory disease space. The path forward requires not only validation of these emerging therapies in larger, multicenter clinical trials but also mechanistic studies aimed at understanding the tumor-immune dynamics specific to UM. A precision immuno-oncology approach, integrating biomarker-driven patient selection, rational combination therapies, and advanced delivery platforms, is likely to be key in unlocking durable responses and improving survival for patients with mUM. While recent advances in uveal melanoma (UM) diagnosis and treatment show significant therapeutic promise, their clinical translation faces substantial limitations. The implementation of these innovations remains constrained by two critical barriers: (1) prohibitively high costs associated with advanced diagnostic technologies and novel therapies, and (2) unequal global accessibility across healthcare systems. These socioeconomic and infrastructural challenges must be urgently addressed through multidisciplinary strategies, including cost-reduction initiatives, healthcare policy reforms, and the development of scalable alternatives, to ensure equitable patient access and maximize the real-world impact of these medical advancements.

## Figures and Tables

**Table 1:** Current immunotherapy options in patients with mUM.

Drugs	References
**ICIs**	
Anti-CTLA-4 single-agent therapy	
Ipilimumab	[24,25,26,27,28,29,30,31,32,33,34,35,36,37,38,39,40,41,42,43,44]
Tremelimumab	[45,46], NCT01034787
Anti-PD-1 single-agent therapy	
Nivolumab	[38,39,47,48,49,50,51,52,53,54]
Pembrolizumab	[38,39,43,49,51,53,54,55,56,57,58,59,60,61]
Spartalizumab	NCT04802876
Anti-PD-L1 single-agent therapy	
Atezolizumab	[59,62]
Avelumab	[63], NCT04328844
Other ICIs	
Anti-TIM-3 (INCAGN02390)	NCT03652077
Targeting-PD-1 and anti-IL-2Rα (REGN10597)	NCT06413680
Combined ICIs	
Combined anti-PD-1 and anti-CTLA-4	[40,49,64,65,66,67,68,69,70,71,72,73,74,75,76,77], NCT06519266
**TCR-based therapy**	
Non-cell-based therapy	
Tebentafusp (IMCgp100)	[18,78,79,80]
Cell-based therapy	
Genetically unmodified T cells (TILs therapy)	[10]
Genetically modified T cells: classical TCR T cells - MART1-specific TCR therapy - PRAME-specific TCR therapy - SLC45A2-specific TCR therapy - MAGE-C2-specific TCR therapy CAR T cells - C7R-GD2-specific CAR-T cells therapy	[11,12]
**Vaccines**	
Cell-based vaccines	[13,14,15]
Peptide-based vaccines	
Nucleic acid-based vaccines	[16,17]
Oncolytic virus-based vaccines	
**Combined therapy (ICIs with…)**	
Chemotherapy: -Lenvatinib -Entinostat, -Nab-paclitaxel -Pemetrexed -Carboplatin -Ruxolitinib -Cisplatin -Obinutuzumab -Melphalan -IOA-244 -LNS8801 -APG-115 -Sitravatinib -Olaparib -ONM-501	NCT05308901, NCT05282901 NCT02697630 NCT05075993, NCT02158520 NCT04993677, NCT04328844 NCT04993677 NCT04328844 NCT04328844 NCT04551352 NCT04283890 NCT04328844 NCT04130516 NCT03611868 NCT05542342 NCT05524935 NCT06022029
Targeted therapy (mAbs): -CD40 agonist (Dacetuzumab, LVGN7409) -CD137 agonist (LVGN6051) -TLR-9 agonist (SD-101) -Anti-VEGF (Bevacizumab, Lenvatinib, Ziv-Aflibercept) -Anti-LAG-3 (Relatlimab) -Anti-Tyrp1 T cell engager (RO7293583) -Anti-IL-6R (Tocilizumab) -Anti-CD20 (Obinutuzumab) -Anti-TNF (Adalimumab) -Anti-tissue factor (ICON-1) -Anti-GD2 (iodine I 131 mAb 3F8)	NCT04993677, NCT05075993 NCT05075993 NCT04935229 NCT05075993, NCT02158520, NCT05308901, NCT05282901 NCT06121180 NCT02519322 NCT04551352 NCT04551352 NCT04551352 NCT04551352 NCT02771340 NCT00445965
SIRT	NCT02913417
Oncolytic viruses	NCT02831933, NCT03408587, NCT06581406
Peptide vaccine	NCT0084656, NCT0032045, NCT0025181
Non-cell-based therapy (Tebentafusp)	NCT02535078
Cell-based therapy (autologous CD8+ SLC45A2- T cells)	NCT03068624

Anti-CTLA-4: anti-cytotoxic T-lymphocyte-associated protein 4; Anti-PD-1: anti-programmed cell death protein 1; Anti-PD-L1: anti-programmed cell death ligand 1; CAR: chimeric antigen receptor; DC: dendritic cell; GD2: disialoganglioside; gp100: glycoprotein 100; ICIs: immune checkpoint inhibitors; ICON-1: human immuno-conjugate 1; Ig: immunoglobulin; IL-6R: interleukin 6 receptor; ITIM: immunoreceptor tyrosine-based inhibitory motif; LAG-3: lymphocyte-activation gene 3; mAb: monoclonal antibodies; MAGE C2: melanoma-associated antigen C2; MART-1: melanoma antigen recognized by T cells 1; PRAME: preferentially expressed antigen in melanoma; SIRT: selective internal radiation therapy; SLC45A2: solute carrier family 45 member 2; TCR: T cell receptor; TIL therapy: adoptive tumor-infiltrating lymphocyte therapy; TIGIT: T cell immunoreceptor with Ig and ITIM domain; TIM-3: T cell immunoglobulin and mucin domain 3; TLR: Toll-like receptor; TNF: tumor necrosis factor; Tyrp1: tyrosinase related protein 1; VEGF: vascular endothelial-derived growth factor.

**Table 3:** Ongoing and completed clinical trials investigating TCR-based therapies in patients with mUM, registered on ClinicalTrials.gov. Both non-cellular and cellular TCR-based approaches are included.

Treatment	Conditions	Phase	Actual Enrollment	Trial Period	Sponsor/Collaborators	Status	NCT No. (Reference)
**Non-cellular TCR-based therapy**							
Tebentafusp + TheraSphere™ Yttrium-90 Trans-Arterial Radioembolization	mUM	II	30	February 2025–February 2031	University of Miami (Miami, FL, USA); Immunocore Ltd. (Abingdon, Oxfordshire, UK)	Not yet recruiting	NCT06627244
Tebentafusp-Tebn	Locally Advanced Unresectable Uveal Melanoma	II	19	January 2025–April 2029	Thomas Jefferson University (Philadelphia, PA, USA)	Not yet recruiting	NCT06414590
Tebentafusp	mUM	II	44	December 2024–November 2029	Diwakar Davar (Pittsburgh, PA, USA); Immunocore Ltd. (Abingdon, Oxfordshire, UK)	Not yet recruiting	NCT06070012
Tebentafusp	UM	III	290	November 2024–November 2032	European Organisation for Research and Treatment of Cancer–EORTC (Brussels, Belgium); Northwell Health (New Hyde Park, NY, USA); Immunocore Ltd. (Abingdon, Oxfordshire, UK)	Recruiting	NCT06246149
Tebentafusp-Tebn + GM-CSF (Sargramostim) + Carmustine (BCNU)	mUM	I/II	109	October 2024–August 2026	Thomas Jefferson University (Philadelphia, PA, USA); Sidney Kimmel Cancer Center at Thomas Jefferson University (Philadelphia, PA, USA)	Not yet recruiting	NCT06626516
Tebentafusp	Melanoma (Skin); UM	II	850	July 2022–June 2026	University of Oxford (Oxford, United Kingdom); Immunocore Ltd. (Abingdon, Oxfordshire, UK); Natera, Inc. (Austin, TX, USA)	Recruiting	NCT05315258
Tebentafusp or Investigator choice (Dacarbazine, Ipilimumab, or Pembrolizumab)	UM	II	378	October 2017–June 2025	Immunocore Ltd. (Abingdon, Oxfordshire, UK)	Active, not recruiting	NCT03070392 [18]
Tebentafusp	Malignant Melanoma	II	3	January 2017–April 2019	Immunocore Ltd. (Abingdon, Oxfordshire, UK)	Terminated	NCT02889861
Tebentafusp	UM	I/II	146	February 2016–October 2022	Immunocore Ltd. (Abingdon, Oxfordshire, UK)	Completed	NCT02570308 [79,80]
Tebentafusp + Durvalumab + Tremelimumab	Malignant Melanoma	Ib/II	0	November 2015–September 2023	Immunocore Ltd. (Abingdon, Oxfordshire, UK); AstraZeneca (Cambridge, UK)	Withdrawn	NCT02535078
Tebentafusp	Malignant Melanoma	I	84	September 2010–February 2017	Immunocore Ltd. (Abingdon, Oxfordshire, UK)	Completed	NCT01211262 [78]
**Cellular TCR-based therapy**							
Autologous TILs + Melphalan + IL-2	UM; mCM	I	6	December 2023–December 2029	Vastra Gotaland Region (Västra Götaland County, Sweden)	Not yet recruiting	NCT05903937
OBX-115 (IL15 expressing TIL)							
Autologous TILs + Melphalan + IL-2	mUM; mCM	I	6	February 2023–February 2030	Vastra Gotaland Region (Västra Götaland County, Sweden); Miltenyi Biomedicine GmbH (Bergisch Gladbach, Germany)	Active, not recruiting	NCT04812470
Autologous TILs (TBio-4101) + Pembrolizumab	UM; CM; Breast Cancer; Colorectal Cancer; Non-Small-Cell Lung Cancer; Head and Neck Squamous Cell Carcinoma	I	60	January 2023–June 2025	Turnstone Biologics, Corp. (Ottawa, ON, Canada)	Recruiting	NCT05576077
Autologous TILs (LN-144/LN-145)	UM; mUM; Melanoma; Metastatic Melanoma	I	20	November 2022–May 2025	Memorial Sloan Kettering Cancer Center (New York, NY, USA); Iovance Biotherapeutics, Inc. (San Carlos, CA, USA)	Recruiting	NCT05607095
Autologous TILs (TBio-4101) + IL-2 + Cyclophosphamide + Fludarabine	Metastatic Melanoma; UM; Acral Melanoma; Mucosal Melanoma	I	25	December 2022–December 2026	H. Lee Moffitt Cancer Center and Research Institute (Tampa, FL, USA); Turnstone Biologics, Corp. (Ottawa, ON, Canada)	Recruiting	NCT05628883
Autologous TILs (LN-144)	Metastatic Melanoma	I	10	November 2022–November 2025	Memorial Sloan Kettering Cancer Center (New York, NY, USA); Iovance Biotherapeutics, Inc. (San Carlos, CA, USA)	Active. Not recruiting	NCT05640193
Autologous MAGE-C2 TCR-T cells + Valproic acid + 5′ azacytide	Melanoma; UM; Head and Neck Cancer	I/II	20	October 2020–October 2027	Erasmus Medical Center (Rotterdam, The Netherlands); Ludwig Institute for Cancer Research (New York, NY, USA); Dutch Cancer Society (Amsterdam, The Netherlands); Stichting Coolsingel Rotterdam grant (Rotterdam, The Netherlands); Jan Ivo Stichting grant (Amsterdam, The Netherlands)	Recruiting	NCT04729543
C7R-GD2.CAR-T cells + Cyclophosphamide + Fludarabine	Relapsed and Refractory Neuroblastoma; Relapsed Osteosarcoma; Relapsed Ewing Sarcoma; Relapsed Rhabdomyosarcoma; UM; Phyllodes Breast Tumor	I	94	April 2019–May 2038	Naylor College of Medicine (Houston, TX, USA); Center for Cell and Gene Therapy, Baylor College of Medicine (Houston, TX, USA); The Methodist Hospital System (Houston, TX, USA); Cancer Prevention Research Institute of Texas (Austin, TX, USA)	Active, not recruiting	NCT03635632
Autologous TILs + IL-2 + Cyclophosphamide + Fludarabine	UM; Uveal Neoplasms	II	47	May 2018–December 2027	Udai Kammula (Bethesda, MD, USA);	Recruiting	NCT03467516
Autologous CD8+ SLC45A2-specific T Lymphocytes + Aldesleukin + Cyclophosphamide+ Ipilimumab	Metastatic Malignant Neoplasm in the Liver; mUM	I	34	September 2017–July 2025	M.D. Anderson Cancer Center (Houston, TX, USA)	Active, not recruiting	NCT03068624
Autologous CD8+ SLC45A2- T cells+ Cyclophosphamide + IL-2 + Ipilimumab	Metastatic Malignant Neoplasm in the Liver; mUM	Ib	34	September 2017–July 2025	MD Anderson Cancer Center (Houston, TX, USA);	Active, not recruiting	NCT03068624
Autologous PRAME TCR-T cells (BPX-701) + Rimiducid + IL-2	Acute Myeloid Leukemia; Myelodysplastic Syndrome; UM	I/II	4	April 2017–July 2020	Bellicum Pharmaceuticals (Houston, TX, USA)	Terminated	NCT02743611 [11]
Pembrolizumab + TIL + IL-2	Metastatic Melanoma; Cutaneous Melanoma	II	18	August 2015–October 2022	M.D. Anderson Cancer Center (Houston, TX, USA);	Completed	NCT02500576
Anti-MAGE-A3-DP4 T Cell Receptor (TCR) Peripheral Blood Lymphocytes (PBL) + Cyclophosphamide + Fludarabine + Aldesleukin	Melanoma	I/II	21	February 2014–March 2021	NCI (Bethesda, MD, USA)	Completed	NCT02111850
Autologous MART-1 TCR-T cells + Cyclophosphamide + Fludarabine	Stage IV Skin Melanoma; Eye Melanoma	I/IIa	12	March 2012–January 2020	The Netherlands Cancer Institute (Amsterdam, The Netherlands)	Unknown	NCT02654821 [12]
Autologous TILs + IL-2 + Cyclophosphamide + Fludarabine	Metastatic Ocular Melanoma; mUM	II	24	March 2013–May 2017	NCI (Bethesda, MD, USA)	Terminated	NCT01814046 [10]
DCs + T cells + Cyclophosphamide + IL-2 + Fludarabine + Mesna	Melanoma	II	1230	February 2006–February 2030	M.D. Anderson Cancer Center (Houston, TX, USA); Prometheus Laboratories (San Diego, CA, USA); Key Biologics, LLC (Memphis, TN, USA); NCI (Bethesda, MD, USA); Adelson Medical Research (Las Vegas, NV, USA)	Active, not recruiting	NCT00338377

CAR: chimeric antigen receptor; DCs: dendritic cells; IL: interleukin; MAGE C2: melanoma-associated antigen C2; MART-1: melanoma antigen recognized by T cells 1; mUM: metastatic uveal melanoma; NCI: National Cancer Institute; PRAME: preferentially expressed antigen in melanoma; SLC45A2: solute carrier family 45 member 2; TCR: T cell receptor; TIL: tumor infiltrating lymphocytes; Recombinant VSV-expressing IFN-b and TYRP1: recombinant vesicular stomatitis virus-expressing INF β and tyrosinase related protein 1.

**Table 4:** Clinical trials using vaccine therapy in mUM patients, registered in ClinicalTrials.gov. The available cancer vaccines for mUM patients are cell-based, peptide-based, nucleic acid-based, and viral-based.

Treatment	Conditions	Phase	Actual Enrollment	Trial Period	Sponsor/Collaborators	Status	NCT No. (Reference)
**Cell-based vaccines**							
Autologous DCs loaded with autologous tumor mRNA encoding for IKKβ	Melanoma; mUM	I	12	June 2020–January 2024	Hasumi International Research Foundation (Bethesda, MD, USA);	Unknown	NCT04335890 [13]
Autologous DCs loaded with autologous tumor mRNA	UM	III	200	June 2014–December 2023	University Hospital Erlangen (Erlangen, Germany), University Hospital Lübeck (Lübeck, Germany), University Hospital Munich (Munich, Germany), Universitätsklinikum Hamburg-Eppendorf (Hamburg, Germany), University Hospital Homburg/Saar (Hamburg, Germany), Universitätsklinikum Köln (Köln, Germany), University Hospital Tuebingen (Tübingen, Germany), University Hospital, Essen (Essen, Germany), Wuerzburg University Hospital (Würzburg, Germany)	Unknown	NCT01983748 [14]
Autologous DCs loaded with autologous tumor mRNA encoding for gp100 and tyrosinase	UM	I/II	23	June 2009–April 2016	Radboud University Medical Center (Nijmegen, Netherlands), Rotterdam Eye Hospital (Rotterdam, Netherlands)	Terminated	NCT00929019 [15]
Autologous DCs loaded with melanoma peptides (MART-1, gp100, tyrosinase)	Intraocular Melanoma; Melanoma (Skin)	II	6	October 2003–June 2005	University of Southern California (Los Angeles, CA, USA); NCI (Bethesda, MD, USA)	Completed	NCT00334776
Autologous DCs loaded with melanoma peptides (MART-1, gp100, and others) + Fludarabine + ALI	Intraocular Melanoma; Melanoma (Skin)	I	18	February 2006–March 2012	H. Lee Moffitt Cancer Center and Research Institute (Tampa, FL, USA); NCI (Bethesda, MD, USA)	Completed	NCT00313508
**Peptide-based vaccines**							
6MHP/NeoAg-mBRAF + Adjuvants (PolyICLC + CDX-1140)	Melanoma; Ocular melanoma; UM	I/II	22	September 2020–March 2024	Craig L Slingluff, Jr (Charlottesville, VA, USA); Celldex Therapeutics (Fall River, MA, USA)	Completed	NCT04364230
6MHP + Adjuvants (Montanide ISA-51 + polyICLC) + CDX-1127	Melanoma	I/II	33	November 2018–January 2024	Craig L Slingluff, Jr (Charlottesville, VA, USA); Celldex Therapeutics (Fall River, MA, USA)	Completed	NCT03617328
MELITAC 12.1 + lipopolysaccharide + polyICLC + Montanide ISA-51	Melanoma	I	53	October 2012–July 2014	Craig L Slingluff, Jr (Charlottesville, VA, USA); University of Virginia (Charlottesville, VA, USA); NCI (Bethesda, MD, USA); Oncovir, Inc. (Washington, DC, USA)	Completed	NCT01585350
MART-1/gp100/tyrosinase (in IFA) + Sargramostim	Ocular Melanoma; Multiple cancers	III	815	February 2000–January 2013	NCI (Bethesda, MD, USA)	Completed	NCT01989572
MELITAC 12.1 peptide + Epacadostat	Mucosal Melanoma; Recurrent Melanoma; Recurrent UM; Skin Melanoma (Stage IIIA-IV); UM (Stage IIIA-IV)	II	11	September 2013–May 2017	Fred Hutchinson Cancer Center (Seattle, WA, USA); Incyte Corporation (Wilmington, DE, USA); NCI (Bethesda, MD, USA); University of Virginia (Charlottesville, VA, USA)	Completed	NCT01961115
Multi-epitope melanoma peptide vaccine + tetanus toxoid helper peptide (in IFA)	Intraocular Melanoma; Malignant Conjunctival Neoplasm; Melanoma (Skin)	I	45	May 2008–June 2009	Craig L Slingluff, Jr (Charlottesville, VA, USA); NCI (Bethesda, MD, USA)	Completed	NCT00705640
MART-1/gp100/tyrosinase (in IFA) + GM-CSF and CpG 7909 (PF3512676)	Intraocular Melanoma; Malignant Conjunctival Neoplasm; Melanoma (Skin)	I	22	October 2008–December 2011	Ahmad Tarhini (Tampa, FL, USA); NCI (Bethesda, MD, USA)	Completed	NCT00471471
gp100/MAGE-3 + Leuprolide	Melanoma	II	98	November 2005–October 2012	M.D. Anderson Cancer Center (Houston, TX, USA)	Completed	NCT00254397
6MHP vaccine + GM-CSF + (in IFA)	Intraocular Melanoma; Melanoma (Skin), Stage IIb-IV	I/II	39	July 2003–May 2006	University of Virginia (Charlottesville, VA, USA); NCI (Bethesda, MD, USA)	Completed	NCT00089219
Multi-epitope melanoma peptide vaccine + GM-CSF (in IFA)	Intraocular Melanoma; Melanoma (Skin), Stage III-IV	II	7	August 2002–November 2005	University of Virginia (Charlottesville, VA, USA); NCI (Bethesda, MD, USA)	Completed	NCT00089206
Multi-epitope melanoma peptide vaccine (gp100/tyrosinase/MAGE-3.1) in IFA + agatolimod sodium	Intraocular Melanoma; Multiple cancers	II	42	May 2004–September 2007	University of Southern California (Los Angeles, CA, USA); NCI (Bethesda, MD, USA)	Completed	NCT00085189
Tyrosinase/gp100/MART-1 Peptide vaccine + Ipilimumab	Intraocular Melanoma; Melanoma (Skin).	II	77	May 2004–October 2009	Bristol-Myers Squibb (New York, NY, USA); NCI (Bethesda, MD, USA)	Completed	NCT00084656
MART-1/gp100/tyrosinase/NA17-A	Intraocular Melanoma.	III	13	February 2002–February 2003	European Organisation for Research and Treatment of Cancer–EORTC (Brussels, Belgium)	Terminated (low accrual)	NCT00036816
gp100 (in IFA) + Ipilimumab	Intraocular Melanoma; Melanoma (Skin), Stage IV	II	not mentioned	January 2002–August 2006	NCI (Bethesda, MD, USA)	Completed	NCT00032045
MART-1/gp100/tyrosinase (in IFA) + IL-12 + GM-CSF + alum adjuvant	Intraocular Melanoma; Melanoma (Skin), Stage II-IV	II	60	February 2002–November 2007	University of Southern California (Los Angeles, CA, USA); NCI (Bethesda, MD, USA)	Completed	NCT00031733
MART-1/gp100/tyrosinase (in IFA) + Ipilimumab	Intraocular Melanoma; Melanoma (Skin), Stage III-IV	I	19	October 2001–June 2005	University of Southern California (Los Angeles, CA, USA); NCI (Bethesda, MD, USA)	Completed	NCT00025181
gp100/MART-1 (in IFA) + IL-2	Extraocular Extension Melanoma; Recurrent Intraocular Melanoma	II	Not mentioned	February 2001–March 2007	NCI (Bethesda, MD, USA)	Completed	NCT00020475
MAGE-12 (in IFA) + IL-2	Melanoma; Eye cancer; Multiple cancers	I	Not mentioned	July 2000–Not mentioned	NCI (Bethesda, MD, USA)	Completed	NCT00020267
MART-1/gp100/tyrosinase (in IFA) + progenipoietin	Intraocular Melanoma; Melanoma (Skin), Stage III-IV	I	15	June 2000–October 2002	University of Southern California (Los Angeles, CA, USA); NCI (Bethesda, MD, USA)	Terminated (Toxicity/Side Effects)	NCT00005841
gp100/tyrosinase (in IFA) + IL-12	Intraocular Melanoma; Melanoma (Skin), Stage III-IV	II	48	November 1998–September 2004	University of Southern California (Los Angeles, CA, USA); NCI (Bethesda, MD, USA)	Completed	NCT00003339
**Nucleic acid-based vaccines**							
Chimeric tyrosinase DNA vaccine using TriGrid Delivery System for i.m. electroporation.	Intraocular Melanoma; Melanoma (Skin)	I	24	April 2007–May 2010	Ichor Medical Systems Incorporated (San Diego, CA, USA); Memorial Sloan Kettering Cancer Center (New York, NY, USA)	Completed	NCT00471133 [16]
Chimeric (mouse) gp100 DNA vaccine by i.m. or PMED.	Intraocular Melanoma; Melanoma (Skin), Stage IIb-IV	I	35	October 2006–March 2011	Memorial Sloan Kettering Cancer Center (New York, NY, USA); NCI (Bethesda, MD, USA)	Completed	NCT00398073 [17]
**Oncolytic virus-based vaccines**							
Nivolumab + RP2/Ipilimumab	mUM	II/III	280	January 2025–October 2031	Replimune Inc. (Woburn, MA, USA)	Recruiting	NCT06581406
RP2 + Nivolumab	Cancer	I	36	October 2019–April 2028	Replimune Inc. (Woburn, MA, USA)	Recruiting	NCT04336241
VSV-IFNβ-TYRP1	mUM; CM; Metastatic Choroid Melanoma; Metastatic Melanoma; Metastatic Mucosal Melanoma	I	12	June 2019–January 2027	Mayo Clinic (Rochester, MN, USA); NCI (Bethesda, MD, USA)	Active, not recruiting	NCT03865212
CVA21 + Ipilimumab	UM; Liver Metastases	Ib	11	January 2018–May 2019	Viralytics (Sydney, Australia)	Completed	NCT03408587
ADV/HSV-tk + Valacyclovir + Nivolumab + SBRT	mUM; Lung Squamous Cell Carcinoma Stage IV; Nonsquamous NSCLC	II	11	February 2017–November 2020	Eric Bernicker, MD (Frisco, CO, USA), The Methodist Hospital System (Houston, TX, USA)	Terminated	NCT02831933

ADV/HSV-tk: adenovirus-mediated expression of herpes simplex virus thymidine kinase; ALI: autologous lymphocyte infusion; CVA21: Coxsackie virus A21; DCs: dendritic cells; GM-CSF: granulocyte-monocyte colony-stimulating factor; GPNMB: glycoprotein nonmetastatic B; IFA: incomplete Freund’s adjuvant; IKKβ: IkB kinase β; IL: interleukin; i.m.: intramuscular; MAGE: melanoma AntiGEn; MART-1: melanoma antigen recognized by T cells 1; mRNA: melanoma RNA; NCI: National Cancer Institute; NSCLC: non-small-cell lung cancer PMED: particle-mediated epidermal delivery; SBRT: stereotactic body radiation therapy; VSV-IFNβ-TYRP1: vesicular stomatitis virus expressing human interferon beta and tyrosinase-related protein 1.

## Data Availability

No new data was generated or analyzed in this study. This review is based on previously published research, and all sources are properly cited in the references section.

## Supplementary Materials

The following supporting information can be downloaded at: https://www.mdpi.com/article/10.3390/jcm14145137/s1, Table S1: Clinical Efficacy of Immune Checkpoint Inhibitor (ICI) Monotherapy in Patients with Metastatic Uveal Melanoma (mUM) [24,25,26,27,28,29,30,31,32,33,34,35,36,37,38,39,40,41,42,43,44,47,48,49,50,51,52,53,54,55,56,57,58,59,60,61,62,63]. Table S2: Efficiency of immune checkpoint inhibitors (ICIs) in combined therapy in mUM patients [40,49,64,65,66,67,68,69,70,71,72,73,74,75,76,77]. Table S3: Efficiency of Tebentafusp therapy in mUM patients [18,78,79,110].

## Abbreviations

The following abbreviations are used in this manuscript:

CAR-T	Chimeric antigen receptor T cells
CM	Cutaneous melanoma
CR	Complete response
DC	Dendritic cell
DCR	Disease control rates
DCR	Disease control rate
GM-CSF	Granulocyte–macrophage colony-stimulating factor
HAVCR2	Hepatitis A virus cellular receptor 2
ICI	Immune checkpoint inhibitor
IDO	Indoleamine 2,3-dioxygenase
IFN-β	Interferon-beta
IFN-γ	Interferon-gamma
IHP	Isolated hepatic perfusion
IL-12	Interleukin-12
IL-2	Interleukin-2
LAG-3	Lymphocyte activation gene-3
MDSCs	Myeloid-derived suppressor cells
mOS	Median overall survival
mPFS	Median progression-free survival
mUM	Metastatic uveal melanoma
NSCLC	Non-small-cell lung cancer
ORR	Objective response rates
ORR	Objective response rate
OS	Overall survival
OSR	Overall survival rates
PD	Progressive disease
PFS	Progression-free survival
PHP	Percutaneous hepatic perfusion
PR	Partial response
RECIST	Response Evaluation Criteria in Solid Tumors
SD	Stable disease
SIRT	Selective internal radiation therapy
TACE	Transarterial chemoembolization
TCR	T cell receptor
TCR-T	TCR-engineered T cells
TDO	Tryptophan 2,3-dioxygenase
TIGIT	T cell immunoreceptor with Ig and ITIM domains
TIL	Tumor-infiltrating lymphocyte
TIM-3	Mucin-domain containing-3
TLR9	Toll-like receptor 9
Tregs	Regulatory T cells
TYRP1	Tyrosinase-related protein 1
UM	Uveal melanoma
VEGF	Vascular endothelial growth factor
VSV	Vesicular stomatitis virus
