# Supplementary material for: Advances and Challenges in Immunotherapy for Metastatic Uveal Melanoma: Clinical Strategies and Emerging Targets
Source: J Clin Med. 2025 Jul 19;14(14):5137. doi: 10.3390/jcm14145137 (PMC12295115; doi:10.3390/jcm14145137)
Supplement: Supplementary file 1 [file jcm-14-05137-s001.zip › jcm-3694422-supplementary.pdf]

Journal of
Clinical Medicine

MDPI

Supplementary Material

Supplementary Table S1. Clinical Efficacy of Immune Checkpoint Inhibitor (ICI) Monotherapy in Patients with Metastatic Uveal Melanoma (mUM). Objective response rate (ORR) is defined as the proportion of patients achieving a complete response (CR) or partial response (PR). Disease control rate (DCR) includes patients with CR, PR, or stable disease (SD). Overall survival (OS) refers to the median time in months from treatment initiation to death due to UM, while progression-free survival (PFS) denotes the median time from treatment initiation to documented disease progression.

Study	Treatment	No of UM patients	ORR %	DCR %	mOS (months)	1-year OSR %	mPFS (months)
	**CTLA-4 inhibitors**						
(1)	Ipilimumab 10 mg/kg	9	0.0	22.2	9	NR	NR
(2)	Ipilimumab	20	5.0	40.0	NR	NR	NR
(3)	Ipilimumab + RFA	18	NR	NR	NR	NR	3.6
(4)	Ipilimumab 3 mg/kg	46	6.5	34.8	5.9	NR	2.9
(5)	Ipilimumab 3 mg/kg	5	0.0	40.0	NR	NR	NR
(6)	Ipilimumab 3 mg/kg	9	0.0	22.2	NR	NR	NR
(7)	Ipilimumab 3 mg/kg	82	4.9	34.1	6	31	3.6
(8) NCT00495066	Ipilimumab 3 and 10 mg/kg	39	5.1	20.5	9.6	NR	NR
(9) NCT00495066	Ipilimumab 3 mg/kg	14	7.1	7.1	5.2	27	NR
(10)	Ipilimumab 3 mg/kg	11	NR	NR	5.7	NR	3.5
(11)	Ipilimumab 10 mg/kg	13	7.7	53.8	NR	NR	NR
(12)	Ipilimumab 3 mg/kg	24	4.2	20.8	9.7	NR	2.8
(13)	Ipilimumab 3 mg/kg	13	7.7	15.4	NR	27	NR
(14) NCT01355120	Ipilimumab 3 mg/kg	34	0.0	47.1	6.8	22	2.8
(15)	Ipilimumab 3 mg/kg	15	NR	NR	3.8	39	NR
(16)	Ipilimumab	22	4.5	54.5	NR	NR	NR
(17)	Ipilimumab	63	1.6	31.7	13.38	52.50	NR
(18)	Ipilimumab	24	0	25	9.9	50	3
(19)	Ipilimumab	20	NR	NR	5	NR	NR
(20)	Ipilimumab 3 mg/kg + RFA	19	0.0	10.5	9.7	NR	NR
(20)	Ipilimumab 10 mg/kg + RFA	19	0.0	5.3	14.2	NR	NR
(21)	Ipilimumab	11	0.0	18.2	NR	NR	NR
	**PD1 inhibitors**						
(22)	Pembrolizumab 2 mg/kg	25	8.0	32.0	3	NR	2.1
(23)	Pembrolizumab 2 mg/kg	8	37.5	50.0	NR	NR	4.5
(24)	Pembrolizumab or nivolumab	14	0	42.9	NR	NR	NR
(25)	Pembrolizumab, nivolumab or atezolizumab	58	3	10	9.5	NR	2.7
(26)	Pembrolizumab 3 or 10 mg/kg	54	3.7	13	7.7	NR	2.6
(27) NCT02359851, NCT02626962, NCT01585194	Pembrolizumab 2 mg/kg or nivolumab 3 mg/kg	17	0.0	11.8	9.6	NR	2.3
(28) NCT01585194, NCT02626962	Pembrolizumab 2 mg/kg	54	5.6	22.2	14	NR	3.1
(16)	Nivolumab	15	25.0	25.0	NR	NR	NR
(28)	Nivolumab 3 mg/kg	32	3.1	18.8	10	NR	2.8
(29)	Nivolumab 3 mg/kg	14	0	35.7	14.5	8.30	2.5
(16)	Pembrolizumab	15	13.3	66.7	NR	NR	NR
(17)	Nivolumab or pembrolizumab	37	0	35.1	13.38	52.50	NR
(18)	Pembrolizumab	43	7	34.9	10.3	38.7	4.8
(30) NCT02697630	Pembrolizumab 200mg + Entinostat 5 mg	29	10.3	41.4	11.5	NR	NR

(31) NCT02359851	Pembrolizumab	5	20.0	60.0	not reached	NR	11
(32)	Nivolumab 3 mg/kg	103	NR	NR	12.6	NR	NR
(33) NCT02673970	Pembrolizumab 2 mg/kg	9	0.0	55.6	11.5	11	4.5
(34)	Nivolumab	14	7.1	42.8	15	NR	2.5
(35) NCT02697630	Pembrolizumab + Entinostat	29	NR	NR	13.4	NR	2.1
(21)	PD-1 inhibitors monotherapy	45	8.9	28.9	NR	NR	NR
(36)	Nivolumab	16	18.8	18.8	10.5	18	5.8
	**PD-L1 inhibitors**						
(37) NCT01772004	Avelumab	16	0	43.8	NR	NR	NR
(38)	Atezolizumab 0.1 to 20 mg/kg or >10 mg/kg every	4	0.0	25.0	NR	NR	NR

IEMBO: immunoembolization; NR: not reported. RFA: radiofrequency ablation.

Supplementary Table S2. Clinical Efficacy of Combination Immune Checkpoint Inhibitor (ICI) Therapy in Patients with Metastatic Uveal Melanoma (mUM). Objective response rate (ORR) is defined as the proportion of patients achieving a complete response (CR) or partial response (PR). Disease control rate (DCR) includes patients with CR, PR, or stable disease (SD). Overall survival (OS) refers to the median duration (in months) from initiation of treatment to death attributable to uveal melanoma, while progression-free survival (PFS) denotes the median time from treatment initiation to documented disease progression.

Study	Treatment	No of UM patients	ORR %	DCR %	mOS (months)	1-year OSR %	mPFS (months)
(28)	Ipilimumab + PD-1 inhibitor	15	13.3	26.7	not reached	NR	NR
(39)	Ipilimumab + Pembrolizumab	9	0.0	55.6	18.4	NR	NR
(40) NCT02626962	Ipilimumab + Nivolumab	50	0	52.0	12.7	NR	3.27
(18)	Ipilimumab + Nivolumab	19	21.1	31.6	18.9	58	3.7
(41)	Ipilimumab + Nivolumab	59	16.9	40.7	16.1	NR	3
(42)	Ipilimumab + Pembrolizumab	8	25.0	75.0	NR	NR	NR
(43)	Ipilimumab + Nivolumab	15	NR	NR	not reached	73	NR
(44)	Ipilimumab + Nivolumab	30	NR	NR	12	NR	NR
(45)	CTLA-4, PD-1 and/or PD- L1	89	11.2	34.8	15	NR	2.7
(46)	Ipilimumab + Nivolumab	94	13.8	40.4	16	NR	2.8
(47) NCT02626962	Ipilimumab + Nivolumab/ Pembrolizumab	54	13.5	65.4	12.7	51.9	3
(48)	Ipilimumab + Nivolumab	33	18.2	51.5	19.1	NR	5.5
(49)	Ipilimumab + Nivolumab	38	NR	NR	14.5	64	NR
(50) NCT02913417	Ipilimumab + Nivolumab + Hepatic artery infusions with Yttrium-90 (90Y) resin microspheres	26	20	68	15	NR	5.5
(51)	Ipilimumab + Nivolumab	47	0	29.8	NR	NR	12.75
(52)	Ipilimumab + Nivolumab + Arginine depleting medication, pegylated arginine deiminase (ADI-PEG 20)	9	0	22.2	8.6	NR	1.5

NR: not reported.

Supplementary Table S3. Clinical Efficacy of Tebentafusp Therapy in Patients with Metastatic Uveal Melanoma (mUM). Objective response rate (ORR) is defined as the proportion of patients achieving a complete response (CR) or partial response (PR). Disease control rate (DCR) includes patients with CR, PR, or stable disease (SD). Overall survival (OS) refers to the median duration (in months) from initiation of treatment to death due to uveal melanoma, while progression-free survival (PFS) indicates the median time from treatment initiation to disease progression.

Study	Treatment	No of UM patients	ORR %	DCR %	mOS (months)	1-year OSR %	mPFS (months)
(53) NCT01211262	Tebentafusp (phase I)	18	16.7	61.1	NR	65	NR

(54,55) NCT02570308	Tebentafusp (phase I)	127	15.8	47.4	16.8	74	7.4
	Tebentafusp (phase II)		4.7	22.8	NR	62	2.8
(56) NCT03070392	Tebentafusp (phase III)	252	9	49.1	21.7	73	3.3

NR: not reported.

References

1. Danielli R, Ridolfi R, Chiarion-Sileni V, Queirolo P, Testori A, Plummer R, Boitano M, Calabrò L, De Rossi C, Giacomo AM Di, et al. Ipilimumab in pretreated patients with metastatic uveal melanoma: Safety and clinical efficacy. *Cancer Immunol Immunother* (2012) **61**:41–48. doi:10.1007/s00262-011-1089-0

2. Khan SA, Callahan M, Postow MA, Chapman PB, Schwartz GK, Dickson MA, D'Angelo SP, Luke JJ, Bluth MJ, Roman RA, et al. Ipilimumab in the treatment of uveal melanoma: the memorial Sloan-Kettering Cancer Center experience. *J Clin Oncol* (2012) **30**:8549–8549.

3. Shaw H, Larkin J, Corrie P, Ellis S, Nobes J, Marshall E, Kumar S, Danson S, Plummer R, Nathan P. Ipilimumab for Advanced Melanoma in an Expanded Access Programme (EAP): Ocular, Mucosal and Acral Subtype UK Experience. *Ann Oncol* (2012) **23**:ix374. doi:10.1016/s0923-7534(20)33704-2

4. Maio M, Sileni VC, Pilla L, Nicoletti SVL, Di Guardo L, Queirolo P, De Galitiis F, Mandala M, Guida M, Ascierto PA. Efficacy and Safety of Ipilimumab in Patients with Pretreated, Ocular Melanoma: Experience from Italian Clinics Participating in the European Expanded Access Programme (EAP). *Ann Oncol* (2012) **23**:ix369–ix370. doi:10.1016/s0923-7534(20)33688-7

5. Khattak MA, Fisher R, Hughes P, Gore M, Larkin J. Ipilimumab activity in advanced uveal melanoma. *Melanoma Res* (2013) **23**:79–81. doi:10.1097/CMR.0b013e32835b554f

6. Wiater K, Witaj T, Mackiewicz J, Kalinka-Warzocha E, Wojtukiewicz M, Szambora P, Falkowski S, Rogowski W, Mackiewicz A, Rutkowski P. Efficacy and safety of ipilimumab therapy in patients with metastatic melanoma: A retrospective multicenter analysis. *Wspolczesna Onkol* (2013) **17**:257–262. doi:10.5114/wo.2013.35785

7. Maio M, Danielli R, Chiarion-Sileni V, Pigozzo J, Parmiani G, Ridolfi R, De Rosa F, Del Vecchio M, Di Guardo L, Queirolo P, et al. Efficacy and safety of ipilimumab in patients with pre-treated, uveal melanoma. *Ann Oncol* (2013) **24**:2911–2915. doi:10.1093/annonc/mdt376

8. Luke JJ, Callahan MK, Postow MA, Romano E, Ramaiya N, Bluth M, Giobbie-Hurder A, Lawrence DP, Ibrahim N, Ott PA, et al. Clinical activity of ipilimumab for metastatic uveal melanoma: A retrospective review of the Dana-Farber Cancer Institute, Massachusetts General Hospital, Memorial Sloan-Kettering Cancer Center, and University Hospital of Lausanne experience. *Cancer* (2013) **119**:3687–3695. doi:10.1002/cncr.28282

9. Kelderman S, Van Der Kooij MK, Van Den Eertwegh AJM, Soetekouw PMMB, Jansen RLH, Van Den Brom RRH, Hospers GAP, Haanen JBAG, Kapiteijn E, Blank CU. Ipilimumab in pretreated metastastic uveal melanoma patients. Results of the Dutch Working group on Immunotherapy of Oncology (WIN-O). *Acta Oncol (Madr)* (2013) **52**:1786–1788. doi:10.3109/0284186X.2013.786839

10. Alexander M, Mellor JD, McArthur G, Kee D. Ipilimumab in pretreated patients with unresectable or metastatic cutaneous, uveal and mucosal melanoma. *Med J Aust* (2014) **201**:49–53. doi:10.5694/mja13.10448

11. Rodriguez JMP, Olza MO de, Codes M, Lopez-Martin JA, Berrocal A, García M, Gurpide A, Homet B, Martin-Algarra S. Phase II study evaluating ipilimumab as a single agent in the first-line treatment of adult patients (Pts) with metastatic uveal melanoma (MUM): The GEM-1 trial. *J Clin Oncol* (2014) **32**:9033.

12. Deo MA. Long-term survival benefit from ipilimumab treatment in metastatic uveal melanoma patients. *J Clin Oncol* (2014) **32**:3060.

13. Zimmer L, Vaubel J, Mohr P, Hauschild A, Utikal J, Simon J, Garbe C, Herbst R, Enk A, Kämpgen E, et al. Phase II DeCOG-study of ipilimumab in pretreated and treatment-naïve patients with metastatic uveal melanoma. *PLoS One* (2015) **10**:1–13. doi:10.1371/journal.pone.0118564

14. Zimmer L, Eigentler TK, Kiecker F, Simon J, Utikal J, Mohr P, Berking C, Kämpgen E, Dippel E, Stadler R, et al. Open-label, multicenter, single-arm phase II DeCOG-study of ipilimumab in pretreated patients with different subtypes of metastatic melanoma. *J Transl Med* (2015) **13**:1–11. doi:10.1186/s12967-015-0716-5

15. Ahmad SS, Qian W, Ellis S, Mason E, Khattak MA, Gupta A, Shaw H, Quinton A, Kovarikova J, Thillai K, et al. Ipilimumab in the real world: The UK expanded access programme experience in previously treated advanced melanoma patients. *Melanoma Res* (2015) **25**:432–442. doi:10.1097/CMR.0000000000000185

16. Itchins M, Ascierto PA, Menzies AM, Oatley M, Lo S, Douraghi-Zadeh D, Harrington T, Maher R, Grimaldi AM, Guminski A. A multireferral centre retrospective cohort analysis on the experience in treatment of metastatic uveal melanoma and utilization of sequential liver-directed treatment and immunotherapy. *Melanoma Res* (2017) **27**:243–250. doi:10.1097/CMR.0000000000000343

17. Mignard C, Deschamps Huvier A, Gillibert A, Duval Modeste AB, Dutriaux C, Khammari A, Avril MF, Kramkimel N, Mortier L, Marcant P, et al. Efficacy of Immunotherapy in Patients with Metastatic Mucosal or Uveal Melanoma. *J Oncol* (2018) **2018**: doi:10.1155/2018/1908065

18. Bol KF, Ellebaek E, Hoejberg L, Bagger MM, Larsen MS, Klausen TW, Køhler UH, Schmidt H, Bastholt L, Kiilgaard JF, et al. Real-world impact of immune checkpoint inhibitors in metastatic uveal melanoma. *Cancers (Basel)* (2019) **11**:1–11. doi:10.3390/cancers11101489

19. Arzu Yaşar H, Turna H, Esin E, Murat Sedef A, Alkan A, Oksuzoglu B, Ozdemir N, Sendur MN, Sezer A, Kılıckap S, et al. Prognostic factors for survival in patients with mucosal and ocular melanoma treated with ipilimumab: Turkish Oncology Group study. *J Oncol Pharm Pract* (2020) **26**:267–272. doi:10.1177/1078155219840796

20. Rozeman EA, Prevoo W, Meier MAJ, Sikorska K, Van TM, Van De Wiel BA, Van Der Wal JE, Mallo HA, Grijpink-Ongering LG, Broeks A, et al. Phase Ib/II trial testing combined radiofrequency ablation and ipilimumab in uveal melanoma (SECIRA-UM). *Melanoma Res* (2019)252–260. doi:10.1097/CMR.0000000000000653

21. Koch EAT, Petzold A, Wessely A, Dippel E, Gesierich A, Gutzmer R, Hassel JC, Haferkamp S, Hohberger B, Kähler KC, et al. Immune checkpoint blockade for metastatic uveal melanoma: Patterns of response and survival according to the presence of hepatic and extrahepatic metastasis. *Cancers (Basel)* (2021) **13**: doi:10.3390/cancers13133359

22. Karydis I, Chan PY, Wheater M, Arriola E, Szlosarek PW, Ottensmeier CH. Clinical activity and safety of Pembrolizumab in Ipilimumab pre-treated patients with uveal melanoma. *Oncoimmunology* (2016) **5**:1–8. doi:10.1080/2162402X.2016.1143997

23. Kottschade LA, McWilliams RR, Markovic SN, Block MS, Bisneto JV, Pham AQ, Esplin BL, Dronca RS. The use of pembrolizumab for the treatment of metastatic uveal melanoma. *Melanoma Res* (2016) **26**:300–303. doi:10.1097/CMR.0000000000000242

24. Piperno-Neumann S, Servois V, Mariani P, Cassoux N, Barnhill R, Rodrigues MJ. Activity of anti-PD1 drugs in uveal melanoma patients. *J Clin Oncol* (2016) **34**:9588.

25. Tsai KK, Shoushtari AN, Munhoz RR, Eroglu Z, Piulats JM, Ott PA, Johnson DB, Hwang J, Daud A, Sosman JA, et al. Efficacy and safety of programmed death receptor-1 (PD-1) blockade in metastatic uveal melanoma (UM). *J Clin Oncol* (2016) **34**:9507.

26. Algazi AP. Clinical outcomes in metastatic uveal melanoma treated with PD-1 and PD-L1 antibodies. *Glob Ecol Biogeogr* (2017) **26**:1386–1397. doi:10.1002/cncr.30258

27. van der Kooij MK, Joosse A, Speetjens FM, Hospers GAP, Bisschop C, de Groot JWB, Koornstra R, Blank CU, Kapiteijn E. Anti-PD1 treatment in metastatic uveal melanoma in the Netherlands. *Acta Oncol (Madr)* (2017) **56**:101–103. doi:10.1080/0284186X.2016.1260773

28. Heppt M V., Heinzerling L, Kähler KC, Forschner A, Kirchberger MC, Loquai C, Meissner M, Meier F, Terheyden P, Schell B, et al. Prognostic factors and outcomes in metastatic uveal melanoma treated with programmed cell death-1 or combined PD-1/cytotoxic T-lymphocyte antigen-4 inhibition. *Eur J Cancer* (2017) **82**:56–65. doi:10.1016/j.ejca.2017.05.038

29. Namikawa K, Takahashi A, Tsutsumida A, Mori T, Motoi N, Jinnai S, Kage Y, Muto Y, Nakano E, Yamazaki N. Nivolumab for patients with metastatic uveal melanoma previously untreated with ipilimumab: A single-institutional retrospective study. *Ann Oncol* (2017) **28**:x114. doi:10.1093/annonc/mdx667.003

30. Jespersen H, Olofsson Bagge R, Ullenhag G, Carneiro A, Helgadottir H, Ljuslinder I, Levin M, All-Eriksson C, Andersson B, Stierner U, et al. Phase II multicenter open label study of pembrolizumab and entinostat in adult patients with metastatic uveal melanoma (PEMDAC study). *Ann Oncol* (2019) **30**:v907. doi:10.1093/annonc/mdz394.068

31. Johnson DB, Bao R, Ancell KK, Daniels AB, Wallace D, Sosman JA, Luke JJ. Response to

anti–PD-1 in uveal melanoma without high-volume liver metastasis. *JNCCN J Natl Compr Cancer Netw* (2019) **17**:114–117. doi:10.6004/jnccn.2018.7070

32. Nathan P, Ascierto PA, Haanen J, Espinosa E, Demidov L, Garbe C, Guida M, Lorigan P, Chiarion-Sileni V, Gogas H, et al. Safety and efficacy of nivolumab in patients with rare melanoma subtypes who progressed on or after ipilimumab treatment: a single-arm, open-label, phase II study (CheckMate 172). *Eur J Cancer* (2019) **119**:168–178. doi:10.1016/j.ejca.2019.07.010

33. Jansen YJL, Seremet T, Neyns B. Pembrolizumab for the treatment of uveal melanoma: A case series. *Rare Tumors* (2020) **12**: doi:10.1177/2036361320971983

34. Namikawa K, Takahashi A, Mori T, Tsutsumida A, Suzuki S, Motoi N, Jinnai S, Kage Y, Mizuta H, Muto Y, et al. Nivolumab for patients with metastatic uveal melanoma previously untreated with ipilimumab: A single-institution retrospective study. *Melanoma Res* (2020) **2014**:76–84. doi:10.1097/CMR.0000000000000617

35. Ny L, Jespersen H, Karlsson J, Alsén S, Filges S, All-Eriksson C, Andersson B, Carneiro A, Helgadottir H, Levin M, et al. The PEMDAC phase 2 study of pembrolizumab and entinostat in patients with metastatic uveal melanoma. *Nat Commun* (2021) **12**:1–10. doi:10.1038/s41467-021-25332-w

36. Tacar SY, Selcukbiricik F, Yilmaz M, Erturk K, Sarici AM, Gulturk I, Ayhan M, Tural D. Nivolumab for metastatic uveal melanoma: a multicenter, retrospective study. *Melanoma Res* (2021) **31**:449–455.

37. Keilholz U, Mehnert JM, Bauer S, Bourgeois HP, Patel MR, Gravenor D, Nemunaitis JJ, Taylor MH, Wyrwicz L, Lee K-W, et al. Avelumab in patients with previously treated metastatic melanoma: Phase 1b results from the JAVELIN Solid Tumor trial. *J Clin Oncol* (2018) **36**:191.

38. Hamid O, Molinero L, Bolen CR, Sosman JA, Muñoz-Couselo E, Kluger HM, McDermott DF, Powderly JD, Sarkar I, Ballinger M, et al. Safety, clinical activity, and biological correlates of response in patients with metastatic melanoma: Results from a phase I trial of atezolizumab. *Clin Cancer Res* (2019) **25**:6061–6072. doi:10.1158/1078-0432.CCR-18-3488

39. Kirchberger MC, Moreira A, Erdmann M, Schuler G, Heinzerling L. Real world experience in low-dose ipilimumab in combination with PD-1 blockade in advanced melanoma patients. *Oncotarget* (2018) **9**:28903–28909. doi:10.18632/oncotarget.25627

40. Piulats Rodriguez JM, De La Cruz Merino L, Espinosa E, Alonso Carrión L, Martin Algarra S, López-Castro R, Curiel García MT, Rodriguez Abreu D, Rullan Iriarte AJ, Berrocal Jaime A. Phase II multicenter, single arm, open label study of nivolumab in combination with ipilimumab in untreated patients with metastatic uveal melanoma (GEM1402.NCT02626962). *Ann Oncol* (2018) **29**:viii443. doi:10.1093/annonc/mdy289.003

41. Heppt M V., Amaral T, Kähler KC, Heinzerling L, Hassel JC, Meissner M, Kreuzberg N, Loquai C, Reinhardt L, Utikal J, et al. Combined immune checkpoint blockade for metastatic uveal melanoma: A retrospective, multi-center study. *J Immunother Cancer* (2019) **7**:1–9. doi:10.1186/s40425-019-0800-0

42. Karivedu V, Eldessouki I, Correa Z, Taftaf A, Zhu Z, Makramalla A, Karim NA. Corrigendum to "Nivolumab and Ipilimumab in the Treatment of Metastatic Uveal Melanoma: A Single-Center Experience." *Case Rep Oncol Med* (2019) **2019**:1–1. doi:10.1155/2019/3868790

43. Hogg D, Monzon JG, Savage J, Skinn B, Ernst S, Song X, McWhirter E, Romeyer F, Smylie M. Canadian cohort expanded-access program of nivolumab plus ipilimumab in advanced melanoma. *Curr Oncol* (2020) **27**:204–214. doi:10.3747/co.27.5985

44. Klemen ND, Wang M, Rubinstein JC, Olino K, Clune J, Ariyan S, Cha C, Weiss SA, Kluger HM, Sznol M. Survival after checkpoint inhibitors for metastatic acral, mucosal and uveal melanoma. *J Immunother Cancer* (2020) **8**:1–6. doi:10.1136/jitc-2019-000341

45. Najjar YG, Navrazhina K, Ding F, Bhatia R, Tsai K, Abbate K, Durden B, Eroglu Z, Bhatia S, Park S, et al. Ipilimumab plus nivolumab for patients with metastatic uveal melanoma: a multicenter, retrospective study. *J Immunother cancer* (2020) **8**:1–9. doi:10.1136/jitc-2019-000331

46. Koch EAT, Petzold A, Wessely A, Dippel E, Erdmann M, Heinzerling L, Hohberger B, Knorr H, Leiter U, Meier F, et al. Clinical determinants of long-term survival in metastatic uveal melanoma. *Cancer Immunol Immunother* (2021) **71**:1467–1477. doi:10.1007/s00262-021-03090-4

47. Piulats JM, Espinosa E, de la Cruz Merino L, Varela M, Alonso Carrión L, Martín-Algarra S, López Castro R, Curiel T, Rodríguez-Abreu D, Redrado M, et al. Nivolumab Plus Ipilimumab for Treatment-Naïve Metastatic Uveal Melanoma: An Open-Label, Multicenter, Phase II Trial by the Spanish Multidisciplinary Melanoma Group (GEM-1402). *J Clin Oncol* (2021) **39**:586–598. doi:10.1200/JCO.20.00550

48. Pelster MS, Gruschkus SK, Bassett R, Gombos DS, Shephard M, Posada L, Glover MS, Simien R, Diab A, Hwu P, et al. Nivolumab and Ipilimumab in Metastatic Uveal Melanoma: Results From a Single-Arm Phase II Study. *J Clin Oncol* (2021) **39**:599–607. doi:10.1200/JCO.20.00605

49. Hodi FS, Chapman PB, Sznol M, Lao CD, Gonzalez R, Smylie M, Daniels GA, Thompson JA, Kudchadkar R, Sharfman W, et al. Safety and efficacy of combination nivolumab plus ipilimumab in patients with advanced melanoma: Results from a North American expanded access program (CheckMate 218). *Melanoma Res* (2021) **1**:67–75. doi:10.1097/CMR.0000000000000708

50. Minor DR, Kim KB, Tong RT, Wu MC, Kashani-Sabet M, Orloff M, Eschelman DJ, Gonsalves CF, Adamo RD, Anne PR, et al. A Pilot Study of Hepatic Irradiation with Yttrium-90 Microspheres Followed by Immunotherapy with Ipilimumab and Nivolumab for Metastatic Uveal Melanoma. *Cancer Biother Radiopharm* (2022) **37**:11–16. doi:10.1089/cbr.2021.0366

51. Salaun H, Saint-ghislain M, Servois V, Garcia A, Matet A, Cassoux N, Mariani P, Piperno-neumann S, Rodrigues M. Nivolumab plus ipilimumab in metastatic uveal melanoma : a real-life , retrospective cohort of 47 patients. (2022)1–7.

52. Kraehenbuehl L, Holland A, Armstrong E, O'Shea S, Mangarin L, Chekalil S, Johnston A, Bomalaski JS, Erinjeri JP, Barker CA, et al. Pilot Trial of Arginine Deprivation Plus Nivolumab and Ipilimumab in Patients with Metastatic Uveal Melanoma. *Cancers (Basel)* (2022) **14**:2638. doi:10.3390/cancers14112638

53. Middleton MR, McAlpine C, Woodcock VK, Corrie P, Infante JR, Steven NM, Jeffry Evans TR, Anthoney A, Shoushtari AN, Hamid O, et al. Tebentafusp, a TCR/Anti-CD3 bispecific fusion protein targeting gp100, potently activated antitumor immune responses in patients with metastatic melanoma. *Clin Cancer Res* (2020) **26**:5869–5878. doi:10.1158/1078-0432.CCR-20-1247

54. Sacco JJ, Carvajal R, Butler MO, Shoushtari AN, Hassel JC, Ikeguchi A, Hernandez-Aya L, Nathan P, Hamid O, Rodriguez JMP, et al. A phase (ph) II, multi-center study of the safety and efficacy of tebentafusp (tebe) (IMCgp100) in patients (pts) with metastatic uveal melanoma (mUM). *Ann Oncol* (2020) **31**:S1442–S1443. doi:10.1016/j.annonc.2020.10.552

55. Sacco J, Carvajal R, Butler M, Shoushtari A, Hassel J, Ikeguchi A, Hernandez-Aya L, Nathan P, Hamid O, Piulats J, et al. Updated survival of patients with previously treated metastatic uveal melanoma who recived Tebentafusp. *J Immunother Cancer 2021;9(Suppl* (2021) **9**:A568.

56. Nathan P, Hassel JC, Rutkowski P, Baurain J-F, Butler MO, Schlaak M, Sullivan RJ, Ochsenreither S, Dummer R, Kirkwood JM, et al. Overall Survival Benefit with Tebentafusp in Metastatic Uveal Melanoma. *N Engl J Med* (2021) **385**:1196–1206. doi:10.1056/nejmoa2103485
